# Expert Consensus Methods In The Humanities - An Exploration of their Potential

**DOI:** 10.12688/f1000research.148726.1

**Published:** 2024-06-28

**Authors:** Charlotte C.S. Rulkens, Rik Peels, Lidwine B. Mokkink, Tamarinde Haven, Lex Bouter

**Affiliations:** 1 Department of Philosophy, Vrije Universiteit Amsterdam, Amsterdam, North Holland, The Netherlands; 2 Faculty of Religion and Theology and Faculty of Humanities, Vrije Universiteit Amsterdam, Amsterdam, North Holland, The Netherlands; 3 African Centre for Epistemology and Philosophy of Science, University of Johannesburg, Auckland Park, Gauteng, South Africa; 4 Department of Methodology, Amsterdam Public Health research institute, Amsterdam, The Netherlands; 5 Department of Epidemiology and Data Science, Amsterdam University Medical Centres, Duivendrecht, North Holland, The Netherlands; 6 Department of Methodology and Statistics, Tilburg School of Social and Behavioural Sciences, Tilburg University, Tilburg, North Brabant, The Netherlands

**Keywords:** Consensus, Consensus methods, Humanities, Methodology, Expertise, Epistemology

## Abstract

**Background:**

Expert consensus methods are regularly used in natural, social, and life sciences. This article explores the potential of applying these methods more frequently in humanities research.

**Methods:**

The authors reviewed literature and applied the philosophical methods of conceptual analysis and conceptual engineering.

**Results:**

This article identifies and describes six main elements of expert consensus methods. It also provides an overview of the different types of expert consensus methods regularly used in the natural, social, and life sciences: Delphi studies, nominal groups, consensus conferences, and Glaser’s state of the art method. Subsequently, each of these types is illustrated by an example from the sciences. The article also presents the potential of and objections to the application of expert consensus methods there. It gives four examples of expert consensus methods that were applied in humanities research, also presented in line with the six elements.

**Conclusions:**

The comparisons and categorization show that, as in the natural, social, and life sciences, expert consensus methods in the humanities can in some instances potentially accelerate the epistemic process and enhance transparency, replicability, diversity, and fair processes. Nevertheless, expert consensus methods need to be fine-tuned to do justice to the unique nature and approaches of the humanities and therefore further research is needed.

## 1. Introduction

In this article, we explore the potential of applying structured and formalised expert consensus methods to reach consensus on diverging topics in research in the humanities. ^1^ An important way to make progress in knowledge and understanding is to gradually, via the exchange of evidence, knowledge, expertise, and critical discussion, reach (more) consensus on an issue. ^2^ However, whether there is consensus on an issue at a certain moment in time can sometimes remain unknown, unclear, or implicit. Expert consensus methods can lift off this inconclusiveness by intentionally and formally pursuing or assessing consensus. These methods are regularly used in natural, social, and life sciences. In linguistics, a field that can arguably be grouped under the humanities, formal expert consensus methods are also regularly applied. Other kinds of valuable expert meetings do take place in various disciplines in the humanities, but the expert consensus methods described in this article differ from those by their procedural and formalised character. Therefore, this article explores the potential of applying these formal expert consensus methods more frequently in the humanities.

Our article is structured as follows. After addressing our methodology (§2) we explain what we mean by ‘consensus’ and ‘expert consensus methods’. We do so by distinguishing between six main elements of consensus methods (§3). After that, we provide an overview of expert consensus methods that are regularly used in the natural, social, and life sciences: Delphi studies, nominal groups, consensus conferences, and Glaser’s state of the art method. We also discuss various examples by describing their operationalization of the six elements (§4). We then turn to the humanities and discuss the potential of and the objections to the application of expert consensus methods in the humanities. This is followed by the presentation of four examples of expert consensus methods that have been applied in humanities research, also presented in accordance with the six elements (§5). Subsequently, we discuss opportunities in humanities research for the implementation of expert consensus methods and present our conclusions (§6).

## 2. Methods

To explore the potential of applying these formal expert consensus methods more frequently in the humanities, we composed an interdisciplinary team of both researchers working with consensus methods in practice, and researchers familiar with research within the domain of the humanities. We subsequently organized a brainstorm session, in which knowledge on the topic was shared from both perspectives. We did an informal literature review and applied the philosophical methods of conceptual analysis ( Daly 2010) and conceptual engineering ( Cappelen, Gendler, Hawthorne 2016). This resulted in a first draft, followed by a second group session and by a final version of the article, which was edited and reviewed by all authors.

## 3. What is consensus and what is an expert consensus method?

Philosophers in social epistemology have reflected on the value and function of interaction and consensus as a means to knowledge ( Kuhn 1962, Habermas 1996). Furthermore, over the past few decades various computational models are designed to assess the dynamics of epistemic communities leading to consensus or dissensus ( Longino 2019, O’Connor *et al*., 2024). Some researchers defend the importance of consensus as an indicator for academic trustworthiness, and the value of consensus studies to assess pressing issues such as climate change ( Oreskes 2021). In this article, we take a more practical approach by presenting means to assess or establish consensus. Before we explore the value of expert consensus methods in the humanities, it is helpful to first define what we mean by ‘consensus’. Ordinary language use of ‘consensus’ might suppose that there is consensus only if a group agrees completely (for 100%) on an issue (see e.g., von der Gracht, 2012). However, also when there is no full consensus and only a large percentage of a group agrees, one can speak of consensus on an issue ( Diamond *et al.* 2014).

Clearly, this entails ambiguity, for when is such a group sufficiently large and who are the experts that belong to that group? Moreover, what counts as ‘sufficient’ consensus may differ from discipline to discipline and from issue to issue. Purely deductive disciplines, such as mathematics and logic, may reach full or almost full consensus on some issues and the bar for consensus may, therefore, be high in those fields. Since the questions addressed in the humanities may leave room for multiple viewpoints that are equally warranted by the evidence base, it can be infeasible or not desirable to get a complete or almost complete consensus. The bar for consensus on such issues could be set lower.

Consensus can take the shape of agreeing that something is true or accurate, but also that something is false, or inaccurate, or unreliable. There can even be consensus that we *do not know* something or that the available evidence does not favour any particular hypothesis. A philosophically informed way to put this is that consensus can take the shape of one out of three doxastic (from the Greek *doxa*, ‘view’) attitudes: joint belief, joint disbelief, and joint suspension of judgment.

Furthermore, consensus can have different objects: experts can agree on the viability of a model, on the predictive power of a theory, on the adequacy of a hypothesis, on what best explains a phenomenon, on what the most suitable method is to study something, on policies for prevention, on definitions, and much more. And once consensus is reached, it is not guaranteed to remain static. It can change over time with emerging new viewpoints or insights deriving from new research.

Whether or not there is consensus on an issue at a certain moment in time often remains implicit or inconclusive. This especially holds when there is no full agreement or when it is not clear whether there is ‘sufficient’ agreement. Expert consensus methods aim to make explicit whether or not there is consensus and what the degree of consensus is. This can be useful when the issue at hand is an important one and when the evidence about the issue does not speak for itself because evidence is lacking, is inconsistent, or its interpretation is not obvious. What expert consensus methods do in such cases can be defined as follows:

**Expert consensus methods** are (i) applied by a *process leader or steering committee*, (ii) to reach consensus or assess the degree of consensus among a group of *experts*, (iii) about an *issue*, (iv) based on pre-set *rules of engagement*, (v) with the aim to deliver useful *output*, (vi) for future *users.* They are characterised by constructive and procedural usage of (dis) agreement and the different arguments that are provided therein, (vi) for future *users.* They are characterised by constructive and procedural usage of (dis) agreement and the different arguments that are provided therein.

Let us briefly clarify the core terms of this definition.

i. A *process leader or steering committee* identifies an *issue* and initiates and designs the expert consensus method. They consider which groups have to be represented in the method and then select the *experts* that are to participate as panellists. The process of how *experts* are selected and by what criteria should be made fully transparent, because expert consensus methods find part of their credibility and validity in how the *process leaders or steering committee* determines who counts as an *expert.* In addition to that, the *process leader or steering committee* prepares the *experts* for the overall process and steps of the expert consensus method and provides the *experts* with *rules of engagement.* These can be adjusted at the instigation of the *experts* before starting the method. The *process leader or steering committee* is also responsible for collecting the data and/or information that is needed to assess the *issue* at hand and ensures every *expert* is provided with the same set of information and/or evidence before participating in the method.
ii. *Experts* are the panellists in the consensus procedure that deliver input in the process of measuring and/or reaching consensus. The nature of their expertise can differ, depending on the *issue* and prospected *output* of the expert consensus method. They can be experts by training and profession, e.g., a physician making a diagnosis or a historian interpreting a source. But they also can be experts on the basis of their experience, e.g. patients’ knowledge about their own illness or students delivering input on their learning experiences at school.
iii. *Issues* are at the centre of the expert consensus method, they are the problem to be solved, the proposals to be considered, or the question to be answered.
iv. *Rules of engagement* are part of the design of the expert consensus method and specify its preconditions. These preconditions can be, but are not limited to, how high the threshold for consensus is (i.e. what ‘sufficient’ agreement is), which areas of disagreement may be retained, how interaction is structured, when to transition into finalising the *output,* or what to do when consensus is not reached. *Experts* may suggest changes and/or improvements to the rules of engagement before engaging as panellists. The *rules of engagement* should be made fully transparent.
v. *Output* can be, but is not limited to, answers to closed questions, guidelines, taxonomies, questionnaires, definitions, quality criteria, and policy advice. ^3^
vi. *Users* are those who have an interest in applying the *output* of the consensus, including but not limited to the experts involved. They can be researchers (starting follow-up projects), the public (making informed decisions), professionals (working according to a guideline), institutions (determining their mission and ambitions and governments (implementing policies).

**Figure 1.**
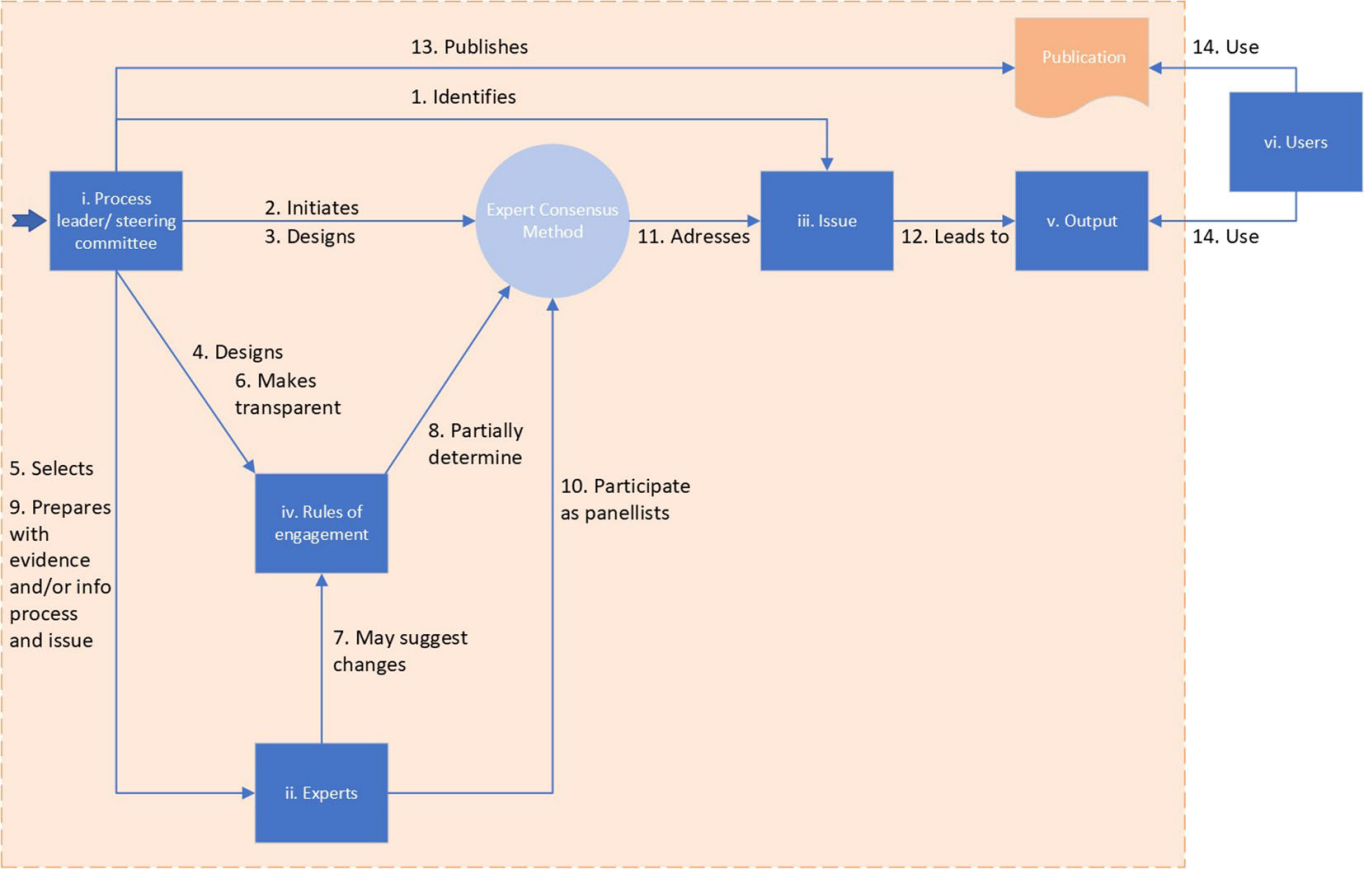
The six elements of expert consensus methods (blue boxes) and the fourteen main steps of the process (arrows).

To successfully apply an expert consensus method, careful preparation by the process leader or steering committee and the effective communication between all participating parties about the process is of vital importance. The roles and responsibilities of different actors (the experts versus the process leader or steering committee) need to be laid out and clearly communicated from the start to enable users to verify the quality of the process. The amount of evidence that is available to the experts determines the part of the issue left about which panellists can reach consensus on: i.e. the inclusion of different viewpoints on the issue is one of the most valuable parts of the process, however, the eventual consensus cannot be in contradiction with the available evidence base. This whole process and its outcomes are made transparent and accessible in a publication by the process leader or steering committee. Figure 1 presents the interrelation of the six elements of expert consensus methods (blue boxes) and the fourteen main steps undertaken in the process (arrows). The boxes and steps that are in the orange field are addressed in the publication and its appendices.

## 4. Types of expert consensus methods

In the natural, social, and life sciences, four distinct expert consensus methods are used frequently: Delphi studies, nominal groups, consensus conferences, and Glaser’s state of the art method. Many studies combine elements of these methods. In Table 1 they are summarised and compared by way of their different characteristics. ^4^ Table 2 shows the actors, factor, and aims of different examples from the natural, social and life sciences. In displaying this, we consider each of the six elements of our earlier definition of ‘expert consensus method’ in paragraph 2.

**Table 1.** Frequently used expert consensus methods in the natural, social, and life sciences and their characteristics.

Name	Delphi study	Nominal group technique	Consensus conference	Glaser’s state of the art method
General description of the process	A series of (online) surveys where panellists are asked to vote and comment on different topics, interspersed by detailed feedback of the survey results which are shared with panellists	Introduction of the topic; silent generation of ideas (panellists write down their own ideas); the facilitator goes round in circles asking one panellist at the time to share their idea, (every idea is recorded) until no further ideas emerge; clarification phase where ideas can be clarified and grouped (seeking explanation); voting and ranking (prioritising the ideas)	Formulation of a list of questions that determine the scope and direction of the conference (questions are widely disseminated); organisation of a conference where all relevant data and views are discussed; presenting draft consensus statement by independent panel; amendments are made immediately	Levelled approach to obtaining consensus. A facilitator gets selected, this person invites a core group, the core group drafts a position paper (multiple iterations); this core group then invited a bigger group of experts to criticize the draft (multiple iterations); the draft is sent out to independent reviewers via a journal leading to revisions of the paper in waves (a certain percentage of invited reviewers submits their reviews, which are then incorporated, to be sent out to new percentage of reviewers) until point of diminishing returns when the paper gets published by the journal
Key references to origins of the method	( Powell 2003) ( Goodman 1987) ( Linstone and Turoff 1975)	( Delbecq *et al.* 1975)	( Stocking *et al.* 1991	( Glaser 1980)
Use of questionnaires (taken from: Black *et al.* 1999)	Yes	No	No	No
Anonymity (taken from: Jones and Hunter 1995)	Yes	Partly (the group is in-person, the voting is anonymous)	No	No
Structured voting	Yes	Yes	Yes (degree of formality depends on sample size)	No
Structured interaction (taken from: McMillan *et al*. 2016)	High	High	Intermediate	Intermediate
Immediate results available (taken from: Potter *et al*. 2004)	Partly	Yes	Partly	No
Co-ownership (experts generally being co-authors of the publication or not)	No	No	Yes	Yes
Sample size (number of experts taking part in the process)	15-100+	2-14	30-3000+	30-50+

**Table 2.** Actors, factors and aims of 4 examples of expert consensus methods in the natural, social, and life sciences.

Method	Delphi study	Nominal Group Technique	Consensus conference	Glaser’s state of the art method
**Publication**	( Brinkman *et al.* 2018)	( Paraskevas 2013)	( Curigliano *et al.* 2017)	( Hodgkin *et al.* 1975)
**Field**	Pharmacology and higher education	Safety research (Anti-terrorism and hospitality management)	Medicine (Oncology, breast cancer)	Medicine (Pulmonary medicine, obstructive airway diseases)
(i) *Process leader or steering committee*	The steering committee consisted of a clinical pharmacologist, a junior doctor, an internist-infectious disease specialist, and a senior lecturer in prescribing	The process leader was a senior lecturer in Strategic Risk Management	The consensus writing committee consisted of 2 chairpersons and 9 researchers and clinicians	The process leader was a behavioural psychologist, the steering committee consisted of 11 physician researcher-practitioners
(ii) *Experts*	The panel consisted of 129 experts from 27 European countries	The panel consisted of 19 hotel security experts as well as members of an international working group on terrorism from 6 European countries	The panel consisted of 42 medical specialists from Europe, North America, Asia and North Africa	The (unstructured) panel consisted of 120 persons who were identified based on their attendance of previous chronic obstructive airway diseases (COPD) key conferences as well as their publications on the issue, or nomination from the steering committee.
(iii) *Issue*	How to modernise and harmonise clinical pharmacology and therapeutics (CPT) education at a European level?	How to best prevent terrorist abusing your hotel, and how to minimise damage?	What is the best way to treat breast cancer in the face of controversies where data from clinical trials is lacking?	What is the status quo of COPD?
(iv) *Rules of engagemen*t	Panellists were asked to rate each outcome (1=very unimportant, 2=unimportant, 3=neutral, 4=important, 5=very important), indicating their agreement that the outcome should be included in the undergraduate CPT curriculum and should be expected of European graduates in order that they can prescribe safely and effectively. If panellists awarded an outcome a score of 4 or 5, they were asked to indicate whether that outcome should be acquired during the preclinical (i.e., bachelor’s degree) or clinical (i.e., master’s degree, clerkships) years of the curriculum, or both	Panellists had to list various security measures for relevance, and were then asked to share their ideas during the workshops, after which they were asked to vote on each proposed measure	Panellists were asked to vote (yes/no/abstain) on a total of 201 different questions related to escalating and de-escalating treatment across subtypes of early-stage breast cancer and treatment types. For some questions, panellists were asked to vote on treatment-specific matters, such as minimal acceptable margins in breast surgery or when the best time is to take a specific biopsy. In cases of a clear difference (88.2% voting “Yes”, 5,9% “No”, and 5,9% “Abstain), the question was transformed into a recommended strategy. For votes close to 50/50, it was reported that the panel was split on the subject and no further recommendations were made	Panellists were asked to read and comment on the current draft and send back their suggested changes
(v) *Output*	Key learning outcomes for undergraduate CPT education in Europe	A six-step baseline anti-terrorism strategy and a series of measures and actions to address the threat of terrorist attacks in hotels	Articulation of strategies for early-stage breast cancer treatment interventions, including guidance on which patients should receive adjuvant chemotherapy, considering also less costly alternatives for countries with limited access to therapeutic and diagnostic resources	Systematised treatment guidelines that can be used to differentiate between different chronic obstructive airway diseases, as well as how the therapeutic program should look, plus the therapeutic modalities, tools, and where application of rehabilitation medicine is useful
(vi) *Users*	Educators, students, health care settings, pharmacologists, patients	Hotel management, anti-terrorism strategists, hotel employees	Clinicians, patients, students, researchers, insurance representatives	Clinicians, patients, students, researchers, insurance representatives

## 5. Expert consensus methods in the humanities, objections, potential and examples

Many expert meetings take place in the humanities as part of discussions at all kinds of conferences and symposia. They are meant to get the input or feedback of experts or to enhance the discussion among experts and are therefore valuable in themselves. However, these meetings are not purposefully designed to formally reach or assess the level of consensus and therefore are not expert consensus methods as defined above. This more formalised approach to expert meetings seems to be rare in the humanities, but might be a valuable addition because of a number of reasons.

First, in formal expert consensus methods, aspects or viewpoints about an issue which normally remain below the surface can be verbalised, shared, and made explicit in the process. It therefore contributes to the articulation of knowledge and understanding of an issue and thus the application of such a method stimulates epistemic progress in itself.

Second, expert consensus methods enhance the transparency and replicability of humanities research because of their well-documented and systemised approach ( Peels and Bouter 2018). ^5^ It can therefore be instrumental to epistemic progress beyond the consensus reached at a moment in time.

Third, expert consensus methods can be helpful in getting a firmer grip on the status quo or state of the art on a particular issue in a field. ^6^ Of course, scholars often already have an idea about that, and such an informed opinion is valuable in itself. However, it can be complemented by a more structured and transparent approach, particularly when the number of scholars involved is large. When applied on a recurrent basis, they might even have the potential to gain insight in how views evolve *over time.*

Fourth, a carefully designed protocol and curated group of experts can foster a fair practice and ensure the consultation of diverse viewpoints. Expert consensus methods provide opportunities to mitigate biases and their ramifications by this cautious selection of experts, but also by blinding certain parts of the process in relation to anonymity of participating experts, for example.

Fifth, beneficial effect of applying expert consensus methods, is that application of its outcomes is often easier after the process of such a method is completed. ^7^

Sixth, expert consensus methods can help the public perceive what the expert consensus on something is Stekelenburg *et al.* (2022) and then to make an informed decision on an issue ( Ordway 2021).

Seventh and finally, making the degree of consensus or lack thereof among a group of experts explicit by way of an expert consensus method can provide guidance for future research by pointing out the gaps in the current evidence base.

But there might also be objections. Research in the humanities takes place within paradigms or schools and can consciously or unconsciously, sometimes may even inevitably, be coloured by the backgrounds and positionality of the researchers. In addition to that, humanities research can be concerned with hermeneutics, i.e. the understanding the values and meanings of texts and other objects. And then there are humanities that have a priori methods, such as ethics, epistemology, and metaphysics. These are methods that do not aim at collecting data, but work with intuitions, principles, values, and thought experiments. Because of these various factors, humanities research sometimes allows for multiple viewpoints, nuances, and interpretations that are all to some extent warranted by the evidence base, sometimes even equally warranted. One might think that this evident complexity of humanities research renders expert consensus methods problematic and/or meaningless.

Yet one of the important purposes of expert consensus methods is to bring various viewpoints, nuances, and interpretations to the surface, so that they can be discussed. In this way, they can contribute to insights into the level of consensus (and lack thereof) on issues *within* certain schools or paradigms or groups with comparable backgrounds, or using the same a priori methods, for example. They can furthermore be valuable for gaining insights on consensus on issues *between* schools or paradigms, groups with different backgrounds, or groups using different a priori methods. Expert consensus methods might even have the potential to increase mutual understanding and provide clarity about what exactly the differences and similarities are. They might be able to make the ‘common ground’ explicit as a result, whilst preventing the domination of certain biases in discussions among experts, for example.

Aside from this, more earthly objections might play a role. Expert consensus methods and their subsequent publication require time and resources, which are rarely in abundance in the humanities. In addition to that, the application of expert consensus methods is not interwoven in the humanities research traditions. Therefore, researchers may not be familiar with the expert consensus methods as defined above, and the advantages it may have for their area of research.

Table 3 presents four examples in which expert consensus methods were applied in humanities research. They are summarised in line with the six elements described in paragraph 2. The examples from the Visual Arts and Museology show that expert consensus methods can be tailor-made to the issue at hand, since the method applied here combined different aspects of the methods presented in Table 1. Aside from these examples, one can think of other potential applications. Establishing consensus and guidelines on the best way to preserve certain historical objects, for example, involving eyewitnesses in the reconstruction of an historical event, or consulting various experts in establishing how to interpret an ancient source. Future case studies might be able to concretise when the application of formal expert consensus methods is useful and when not.

**Table 3.** Examples of expert consensus methods in the humanities.

Expert consensus method	Delphi study	Nominal group	Mixed and tailor-made method that somewhat resembles a consensus conference	Mixed and tailor-made method that somewhat resembles a Glaser’s state of the art method
**Publication**	( Lechner *et al.* 2023)	( Dragouni and Lekakis 2023)	( Rulkens *et al.* 2022-2023)	( *Standing Committee for the Museum Definition – ICOM Define Final Report (2020-2022),* 2022) and ( *ICOM Webinar Defining the Museum in Times of Change: A Way Forward,* 2020)
**Field**	Philosophy (Epistemology)	Conservation (Heritage management)	Visual Arts (Art History)	Museology
(i) *Process leaders or steering committee*	Authors of the paper	Authors of the paper	Multidisciplinary group of researchers	International Council of Museums (ICOM)
(ii) *Experts*	46 Researchers on epistemic responsibilities and/or university assessment instruments and/or (former) administrators with practical knowledge and hands-on experience in leading a university	32 Representatives of local government; academic researchers and members of cultural associations, artists, and residents	4 Rembrandt scholars with various kinds of expertise in art history and technical research into paintings	126 National Committees of museum professionals that are members of ICOM, (precise number of experts unknown)
(iii) *Issue*	What are the core epistemic responsibilities of universities?	What are solutions/actions and/or tactics to protect the rural monuments of the island of Naxos?	Are two paintings painted by Rembrandt, partially painted by Rembrandt or not painted by Rembrandt?	What should count as an international definition of the museum?
(iv) *Rules of engagemen*t (consensus threshold)	Three-round online survey, alternating between closed questions to gain consensus, and open questions to let experts motivate their answers. Panellists rated their agreement to consensus questions on a 5-point Likert scale (strongly agree – somewhat agree – neither agree nor disagree – somewhat disagree – strongly disagree). Consensus defined as 67% (i.e., two-thirds) of panellists (strongly) agreeing to a question. If no consensus was reached, the steering committee made a final decision	Panellists were divided in 5 groups which each met for 2 hours. Five main stages were completed: (i) presentation of problem-issue (ii) ideas sharing and generation and recording, (iii) discussion and listing, (iv) voting and (v) vote counting and conclusions. Consensus was establishes through majority and the qualitative generated data was thematically analysed	Panellists individually assessed the consensus questions and filled in a form in which they could address in percentages the extend in which they were sure about their answers. Subsequently, they were each interviewed to ask for motivations. This was followed by a focus group discussion, led by a chair. The experts then filled in a second form to examine if answers changed due to the group discussion. This was followed by an interview to ask about their motivations. The method ended with a joint debriefing. Qualitative data was thematically analysed. Consensus was defined as 75%, i.e. when three out of four experts (three-quarters) agreed to a question	Committees each shared max 100 keywords for the definition, after discussion each committee submitted max 20 keywords for the definition. Then the keywords were analysed externally and the results were published. Committees reviewed the published results and submitted comments. They could add up to 3 new keywords and comment on keywords they could not accept. A final list of keywords was compiled. Based on this, a small group of special define members submitted 14 proposals for the definition. 5 Were selected by them for publication. Committees identified their preferred proposal. This final proposal was published and put to vote during an international extraordinary general assembly of ICOM members
(v) *Output*	6 core epistemic responsibilities of universities	22 ideas reflecting current needs and proposing responding actions to safeguarding the future of the rural heritage of the island of Naxos	Full consensus amongst panellists about the attribution of two 17 ^th^ century paintings	An internationally supported definition of the museum
(vi) *Users*	Higher education policymakers and university leadership	Heritage experts, managers of rural resources, local communities	Specialists of 17 ^th^ century Dutch paintings, the museums that own these paintings, museum visitors	Museums, museum professionals, policymakers and governments world-wide

## 6. Conclusion

When it comes to expert consensus methods, the sciences and the humanities show more similarities than one might initially think. The humanities lend themselves better to structured expert consensus methods than their subject matter and research methods might suggest. It turns out, that when research questions or aims are alike, similar expert consensus methods could be applied in line with the same six elements presented in this article. Expert consensus methods can in particular instances potentially accelerate epistemic process, enhance transparency, increase replicability, stimulate diversity, and encourage fair processes in humanities research and the application of its findings. An in-depth systematic review of the use of expert consensus methods in the humanities was not part of this study, and therefore other forms of formal expert consensus methods in the humanities may have stayed under the radar. Such an overview could be useful for future exploration of the application of expert consensus methods in the humanities. It would be valuable to further explore the possibilities and limitations of these methods and to investigate how expert consensus methods need to be fine-tuned to do justice to the unique nature and approaches of the humanities. Therefore, it should be a priority to carry out more case studies in the humanities in which a consensus method is used and its feasibility and usefulness are evaluated. Humanities researchers could join forces with researchers from other domains who have experience in designing and carrying out these procedures and learn from each other in the process.

## Author contributions

Charlotte C.S. Rulkens and Rik Peels delivered the overall concept and text for this article, which Lidwine B. Mokkink, Tamarinde Haven and Lex Bouter critically read and revised. In addition to that, Tamarinde Haven and Lidwine B. Mokkink were responsible for Tables 1 and 2.

## Data Availability

Data sharing is not applicable to this article as no datasets were generated or analysed during the current study.

## Footnotes

^1^
The division between the natural sciences, social sciences, life sciences and the humanities can be diffuse, and in many studies, there are various degrees of inter- and multi- disciplinarity between those four groups. For this paper, we count research as part of the humanities when related to one or more of the following disciplines: History; Language and Linguistics; Archeology; Classics; Conservation; History and Philosophy of Science; Literature and Literary Theory; Museology; Music; Philosophy; Theology and Religious studies; Visual Arts and Performing Arts.

^2^
Thus also Grimm, Peels, and Van Woudenberg (forthcoming); Peels (2020).

^3^
See, for example, Paraskevas (2013) and Curigliano *et al.* (2017) mentioned in Table 1.

^4^

Table 1 synthesises criteria as previously discussed in Jones and Hunter (1995), Black *et al.* (1999), Blazey *et al.* (2021).

^5^
Both desiderata lately received much attention. For replicability, see Peels (2019).

^6^
See, for example, Hodgkin *et al.* (1975) mentioned in Table 2.

^7^
One example where this was carried out as part of an issue in the humanities, is the procedure that was successfully completed in 2022 in order to establish a new international museum definition, mentioned in Table 3. An 18-month procedure with hundreds of museum professionals from 126 National Committees from all over the world led to the adoption of a newly proposed definition approved by 92% of the votes. See *Standing Committee for the Museum Definition – ICOM Define Final Report (2020-2022)* (2022) and *ICOM Webinar Defining the Museum in Times of Change: A Way Forward* (2020).
