# Expert Consensus Methods In The Humanities: An Exploration of their Potential

**DOI:** 10.12688/f1000research.148726.2

**Published:** 2024-12-16

**Authors:** Charlotte C.S. Rulkens, Rik Peels, Lidwine B. Mokkink, Tamarinde Haven, Lex Bouter

**Affiliations:** 1 Department of Philosophy, Vrije Universiteit Amsterdam, Amsterdam, North Holland, The Netherlands; 2 Faculty of Religion and Theology and Faculty of Humanities, Vrije Universiteit Amsterdam, Amsterdam, North Holland, The Netherlands; 3 African Centre for Epistemology and Philosophy of Science, University of Johannesburg, Auckland Park, Gauteng, South Africa; 4 Department of Methodology, Amsterdam Public Health research institute, Amsterdam, The Netherlands; 5 Department of Epidemiology and Data Science, Amsterdam University Medical Centres, Duivendrecht, North Holland, The Netherlands; 6 Department of Methodology and Statistics, Tilburg School of Social and Behavioural Sciences, Tilburg University, Tilburg, North Brabant, The Netherlands

**Keywords:** Consensus, Consensus methods, Humanities, Methodology, Expertise, Epistemology

## Abstract

**Background:**

Despite the significant role of consensus and dissensus in knowledge production, formal approaches to consensus are notably less common in the humanities compared to their frequent application in natural, social, and life sciences. This article therefore explores the potential of expert consensus methods in humanities-related research.

**Methods:**

In order to do so, an interdisciplinary team of both sciences researchers experienced in consensus methods and researchers familiar with the domain of the humanities and epistemology, conducted a literary review and exchanged their expertise in multiple brainstorm sessions.

**Results:**

This resulted in the identification of six key elements of expert consensus methods. It also provided for an overview of different types of expert consensus methods that regularly used in the natural, social, and life sciences: Delphi studies, nominal groups, consensus conferences, and Glaser’s state of the art method and illustrative examples from both sciences and humanities-related studies. An overview of possible purposes for applying these methods is provided to identify the research contexts in which these methods have proven their value, which can be extrapolated to humanities related issues for which these methods seem promising.

**Conclusions:**

The comparisons and categorisation show that, when focusing on the purposes, there seem to be humanities-related issues that may lend themselves better to structured expert consensus methods than their subject matter and research methods might suggest. When deliberately applied in context chosen by researchers with expertise in a specific humanities domain, expert consensus methods can accelerate epistemic process, enhance transparency, increase replicability, stimulate diversity, and encourage fair processes in humanities research and the application of its findings.

## 1. Introduction

An important way to make progress in knowledge and understanding is to gradually, via the exchange of evidence, expertise, and critical discussion, reach (more) consensus on a topic. However, whether there is consensus on a certain matter at a moment in time can sometimes remain unknown, be unclear, or remain implicit. Expert consensus methods can lift off this inconclusiveness by intentionally and formally pursuing or assessing consensus. These methods are regularly used in natural, social, and life sciences. Despite the significant role of consensus in the development of (academic) knowledge and understanding (e.g. Kuhn 1962, Habermas 1996, thus also Peels 2020, Grimm *et al. forthcoming*), formal expert consensus methods have hardly been explored within in the context of the humanities. ^1^ Other kinds of valuable expert meetings do take place in various disciplines in the humanities. They usually do not seek to reach a consensus, but to explore a topic in-depth by bringing the relevant experts to the table. Moreover, expert consensus methods described in this article differ from the expert meetings that already take place in the humanities by the former’s procedural and formalised character.

In this paper, we aim to highlight to scholars, particularly those in the humanities, what is possible in terms of a systematic approach to academic consensus. In doing so, we aim to initiate exploration of and discussion about the potential of applying such an approach. What would be the added value of applying expert consensus meetings to humanities-related issues? To achieve this, we first explicate our method (§2), before we provide a framework for the application of expert consensus methods by introducing six key elements (§3). Subsequently, we present common types of expert consensus methods utilised in the natural, social, and life sciences. We highlight their proven value across various types of research through relevant examples and their respective objectives (§4). We then consider both the potential benefits and possible limitations to applying these methods within humanistic research (§5), and conclude with suggestions for future research (§6).

## 2. Methods

To explore the potential of applying expert consensus methods in the humanities, we composed an interdisciplinary team of both researchers from the sciences using consensus methods in practice (LM: epidemiology; TH: qualitative research methodology; LB: epidemiology and data science, research integrity), and researchers familiar with the domain of the humanities and epistemology (CR: art history, museology; RP: epistemology and philosophy of science, religion and theology). In a first brainstorm session, knowledge on the topic was shared from both perspectives. This resulted in a list of consensus methods and examples used in the sciences, drafted by LM and TH. CR carried out a literature search in order to find examples thereof that were applied in her domain. A first draft was written and discussed in a second group session and culminated in a final version, which was edited and reviewed by all authors.

## 3. Six Key elements of expert consensus methods

Philosophers have reflected on the value and function of consensus as a means to knowledge (e.g. Kuhn 1962, Habermas 1996). Some researchers defend the importance of consensus as an indicator for academic trustworthiness, and the value of consensus studies to assess pressing issues such as climate change ( Oreskes 2021). Within the field of personality psychology, others have stressed the importance of fostering and explicating consensus on key preconditions and future research goals to move the entire field forward (Leising *et al.* 2022a, 2022b). In this article, we take a more practical approach by presenting means to assess or establish consensus. Before we explore the value of expert consensus methods in the humanities, it is helpful to first define what we mean by ‘consensus’. Ordinary language use of ‘consensus’ might suppose that there is consensus only if a group agrees completely (for 100%) on an issue (see e.g., von der Gracht *et al.* 2012). However, also when there is no full consensus and only a large percentage of a group agrees, one can speak of consensus on an issue ( Diamond *et al.* 2014).

Clearly, this entails ambiguity, for when is such a group sufficiently large and who are the experts that belong to that group? Moreover, what counts as ‘sufficient’ consensus may differ from discipline to discipline and from issue to issue. Purely deductive disciplines, such as mathematics and logic, may reach full or almost full consensus on some issues and the bar for consensus may, therefore, be high in those fields. Since the questions addressed in the humanities may leave room for multiple viewpoints that are equally warranted by the evidence base, it can be infeasible or not desirable to get a complete or almost complete consensus. The bar for consensus on such issues could be set lower.

Consensus can take the shape of agreeing that something is true or accurate, but also that something is false, or inaccurate, or unreliable. There can even be consensus that we *do not know* something or that the available evidence does not favour any particular hypothesis. A philosophically informed way to put this is that consensus can take the shape of one out of three doxastic (from the Greek *doxa*, ‘view’) attitudes: joint belief, joint disbelief, and joint suspension of judgment (Peels 2017, chapter 1).

Furthermore, consensus can have different objects: experts can agree on the viability of a model, on the predictive power of a theory, on the adequacy of a hypothesis, on what best explains a phenomenon, on what the most suitable method is to study something, on policies for prevention, on definitions, and much more. And once consensus is reached, it is not guaranteed to remain static. It can change over time with emerging new viewpoints or insights deriving from new research.

Whether or not there is consensus on an issue at a certain moment in time often remains implicit or inconclusive. This especially holds when there is no full agreement or when it is not clear whether there is ‘sufficient’ agreement. Expert consensus methods aim to make explicit whether or not there is consensus and what the degree of consensus is. This can be useful when the issue at hand is an important one and when evidence is lacking, it is inconsistent, or its interpretation is not obvious. What expert consensus methods do in such cases can be defined as follows:

**Expert consensus methods** are (i) applied by a *process leader or steering committee*, (ii) to reach consensus or assess the degree of consensus among a group of *experts*, (iii) about an *issue*, (iv) based on pre-set *rules of engagement*, (v) with the aim to deliver useful *output*, (vi) for future *users.* They are characterised by constructive and procedural usage of (dis)agreement and the different arguments that are provided therein.

Let us briefly clarify the core terms of this definition.

i. A *process leader or steering committee* identifies an *issue* and initiates and designs the expert consensus method. They consider which groups have to be represented in the method and then select the *experts* that are to participate as panellists. The process of how *experts* are selected and by what criteria should be made fully transparent, because expert consensus methods find part of their credibility and validity in how the *process leaders or steering committee* determines who counts as an *expert.* In addition to that, the *process leader or steering committee* prepares the *experts* for the overall process and steps of the expert consensus method and provides the *experts* with *rules of engagement.* These can be adjusted at the instigation of the *experts* before starting the method. The *process leader or steering committee* is also responsible for collecting the data and/or information that is needed to assess the *issue* at hand and ensures every *expert* is provided with the same set of information and/or evidence before participating in the method.
ii. *Experts* are the panellists in the consensus procedure that deliver input in the process of measuring and/or reaching consensus. The nature of their expertise can differ, depending on the *issue* and prospected *output* of the expert consensus method. They can be experts by training and profession, e.g., a physician making a diagnosis or a historian interpreting a source. But they also can be experts on the basis of their experience, e.g. patients’ knowledge about their own illness or students delivering input on their learning experiences at school.
iii. *Issues* are at the centre of the expert consensus method, they are the problem to be solved, the proposals to be considered, or the question to be answered.
iv. *Rules of engagement* are part of the design of the expert consensus method and specify its preconditions. These preconditions can be, but are not limited to, how high the threshold for consensus is (i.e. what ‘sufficient’ agreement is), which areas of disagreement may be retained, how interaction is structured, when to transition into finalising the *output,* or what to do when consensus is not reached. *Experts* may suggest changes and/or improvements to the *rules of engagement* before engaging as panellists. The *rules of engagement* should be made fully transparent.
v. *Output* can be, but is not limited to, answers to closed questions, guidelines, taxonomies, questionnaires, definitions, quality criteria, and policy advice.
vi. *Users* are those who have an interest in applying the *output* of the consensus, including but not limited to the experts involved. They can be researchers (starting follow-up projects), the public (making informed decisions), professionals (working according to a guideline), institutions (determining their mission and ambitions and governments (implementing policies).

**Figure 1.**
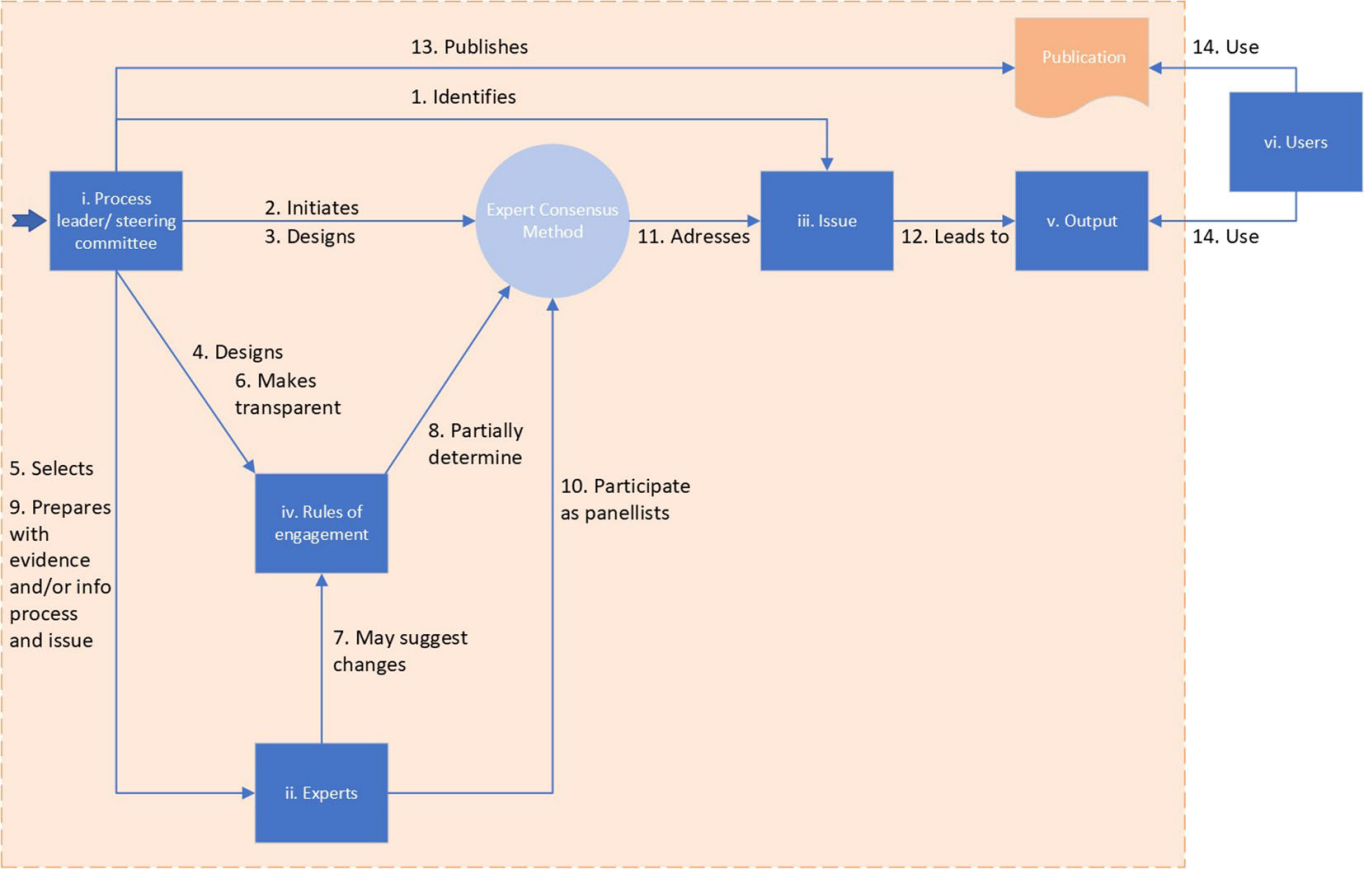
The six elements of expert consensus methods (blue boxes) and the fourteen main steps of the process (arrows). All steps in the orange area are described in the final publication.

To successfully apply an expert consensus method, careful preparation by the process leader or steering committee and the effective communication between all participating parties about the process is of vital importance. The roles and responsibilities of different actors (the experts versus the process leader or steering committee) need to be laid out and clearly communicated from the start to enable users to verify the quality of the process. The amount of evidence that is available to the experts determines the part of the issue left about which panellists can reach consensus on: i.e. the inclusion of different viewpoints on the issue is one of the most valuable parts of the process, however, the eventual consensus cannot be in contradiction with the available evidence base. This whole process and its outcomes are made transparent and accessible in a publication by the process leader or steering committee. Figure 1 presents the interrelation of the six elements of expert consensus methods (blue boxes) and the fourteen main steps undertaken in the process (arrows). The boxes and steps that are in the orange field are addressed in the publication and its appendices.

Naturally, the six elements can be specified by the steering committee. If they deem it valuable to include an independent moderator or to incorporate a member with a critical or divergent perspective—especially if the topic is highly controversial—this remains within their discretion. Additionally, it is up to them to decide who will be listed as co-authors, whether that includes only the steering committee, all participants, or a broader group representation such as the panellists as a whole, for instance.

## 4. Types, examples and purposes

After establishing this foundational understanding of consensus and the dynamic of expert consensus methods, Table 1 presents and compares the four expert consensus methods that are used frequently in the natural, social and life sciences: Delphi studies, nominal groups, consensus conferences, and Glaser’s state of the art method. As expert consensus methods can be tailor-made to the issue at hand, many studies combine elements of these methods. Tables 2 and 3 each display four examples of studies employing one or more of these expert consensus methods, both in the natural, social and life sciences and in humanities-related fields. All examples are described by the six key elements as defined by us.

The subsequent question would be to determine the contexts in which this approach to consensus would prove most valuable. Research in both the humanities and the sciences spans a wide array of disciplines and subdisciplines, with a vast range of variation in types of research and methods, even within the most specialised areas. Classification of research into one of these domains is often gradual in nature, especially since interdisciplinary research increasingly combines approaches traditionally linked to the ‘humanities’ or ‘sciences’. Making general statements on the types of research in which expert consensus methods might be valuable related to the ‘humanities’ versus the ‘sciences’ is therefore difficult. We decided to examine objectives for employing consensus methods in various contexts in which they have demonstrated their value. This could contribute to a directory of instances in which these methods may prove useful in humanities-related fields. Gattrell *et al.* (2024, Table 2) present examples of these objectives in context of the health sciences, such as the establishment of clinical practice guidelines, diagnostic guidelines or reporting guidelines but also the classification of diseases, the setting research priorities, or the formulation of policy. These aims are summarised, adjusted and extended in Table 4 in a manner that enhances their generalizability to other fields of research and were included in Tables 2 and 3. Although these examples highlight specific applications, we think that there is a broader potential for its use in the humanities. For instance, in establishing consensus and guidelines on the best way to preserve certain historical objects (guidelines/policy development), involving eyewitnesses in the reconstruction of an historical event (current state of knowledge), or consulting various experts in establishing how to interpret an ancient source (current state of knowledge). Future research and case studies exploring the potential applications outlined in Table 4 could provide insights into specific instances where the pursuit of formal dis- or consensus may prove to be effective.

**Table 1.** Frequently used expert consensus methods in the natural, social, and life sciences and their characteristics. Synthesises of criteria as previously discussed in Jones and Hunter (1995), Black *et al.* (1999) and Blazey *et al.* (2022).

Name	Delphi study	Nominal Group Technique	Consensus conference	Glaser’s state of the art method
General description of the process	A series of (online) surveys where panellists are asked to vote and comment on different topics, interspersed by detailed feedback of the survey results which are shared with panellists	Introduction of the topic; silent generation of ideas (panellists write down their own ideas); the facilitator goes round in circles asking one panellist at the time to share their idea, (every idea is recorded) until no further ideas emerge; clarification phase where ideas can be clarified and grouped (seeking explanation); voting and ranking (prioritising the ideas)	Formulation of a list of questions that determine the scope and direction of the conference (questions are widely disseminated); organisation of a conference where all relevant data and views are discussed; presenting draft consensus statement by independent panel; amendments are made immediately	Levelled approach to obtaining consensus. A facilitator gets selected, this person invites a core group, the core group drafts a position paper (multiple iterations); this core group then invited a bigger group of experts to criticise the draft (multiple iterations); the draft is sent out to independent reviewers via a journal leading to revisions of the paper in waves (a certain percentage of invited reviewers submits their reviews, which are then incorporated, to be sent out to new percentage of reviewers) until point of diminishing returns when the paper gets published by the journal
Key references to origins of the method	( Powell 2003) ( Goodman 1987) ( Linstone and Turoff 1975) ( Dalkey and Helmer 1963)	( Delbecq *et al.* 1975)	( Stocking *et al.* 1991)	( Glaser 1980)
Use of questionnaires (taken from: Black *et al.* 1999)	Yes	No	No	No
Anonymity (taken from: Jones *et al.* 1995)	Yes	Partly (the group is in-person, the voting is anonymous)	No	No
Structured voting	Yes	Yes	Yes (degree of formality depends on sample size)	No
Structured interaction (taken from: McMillan *et al*. 2016)	High	High	Intermediate	Intermediate
Immediate results available (taken from: Potter *et al*. 2004)	Partly	Yes	Partly	No
Co-ownership (experts generally being co-authors of the publication or not)	No	No	Yes	Yes
Sample size (number of experts taking part in the process)	15-100+	2-14	30-3000+	30-50+

**Table 2.** Four examples of expert consensus methods in the natural, social, and life sciences described by type and according to the six elements.

Method	Delphi study	Nominal Group Technique	Consensus conference	Glaser’s state of the art method
**Publication**	( Brinkman *et al.* 2018)	( Paraskevas 2013)	( Curigliano *et al.* 2017)	( Hodgkin *et al.* 1975)
**Field**	Pharmacology and higher education	Safety research (Anti-terrorism and hospitality management)	Medicine (Oncology, breast cancer)	Medicine (Pulmonary medicine, obstructive airway diseases)
(i) *Process leader or steering committee*	The steering committee consisted of a clinical pharmacologist, a junior doctor, an internist-infectious disease specialist, and a senior lecturer in prescribing	The process leader was a senior lecturer in Strategic Risk Management	The consensus writing committee consisted of 2 chairpersons and 9 researchers and clinicians	The process leader was a behavioural psychologist, the steering committee consisted of 11 physician researcher-practitioners
(ii) *Experts*	The panel consisted of 129 experts from 27 European countries	The panel consisted of 19 hotel security experts as well as members of an international working group on terrorism from 6 European countries	The panel consisted of 42 medical specialists from Europe, North America, Asia and North Africa	The (unstructured) panel consisted of 120 persons who were identified based on their attendance of previous chronic obstructive airway diseases (COPD) key conferences as well as their publications on the issue, or nomination from the steering committee.
(iii) *Issue*	How to modernise and harmonise clinical pharmacology and therapeutics (CPT) education at a European level?	How to best prevent terrorist abusing your hotel, and how to minimise damage?	What is the best way to treat breast cancer in the face of controversies where data from clinical trials is lacking?	What is the status quo of COPD?
(iv) *Rules of engagemen*t	Panellists were asked to rate each outcome (1=very unimportant, 2=unimportant, 3=neutral, 4=important, 5=very important), indicating their agreement that the outcome should be included in the undergraduate CPT curriculum and should be expected of European graduates in order that they can prescribe safely and effectively. If panellists awarded an outcome a score of 4 or 5, they were asked to indicate whether that outcome should be acquired during the preclinical (i.e., bachelor’s degree) or clinical (i.e., master’s degree, clerkships) years of the curriculum, or both	Panellists had to list various security measures for relevance, and were then asked to share their ideas during the workshops, after which they were asked to vote on each proposed measure	Panellists were asked to vote (yes/no/abstain) on a total of 201 different questions related to escalating and de-escalating treatment across subtypes of early-stage breast cancer and treatment types. For some questions, panellists were asked to vote on treatment-specific matters, such as minimal acceptable margins in breast surgery or when the best time is to take a specific biopsy. In cases of a clear difference (88.2% voting “Yes”, 5,9% “No”, and 5,9% “Abstain), the question was transformed into a recommended strategy. For votes close to 50/50, it was reported that the panel was split on the subject and no further recommendations were made	Panellists were asked to read and comment on the current draft and send back their suggested changes
(v) *Output*	Key learning outcomes for undergraduate CPT education in Europe	A six-step baseline anti-terrorism strategy and a series of measures and actions to address the threat of terrorist attacks in hotels	Articulation of strategies for early-stage breast cancer treatment interventions, including guidance on which patients should receive adjuvant chemotherapy, considering also less costly alternatives for countries with limited access to therapeutic and diagnostic resources	Systematised treatment guidelines that can be used to differentiate between different chronic obstructive airway diseases, as well as how the therapeutic program should look, plus the therapeutic modalities, tools, and where application of rehabilitation medicine is useful
(vi) *Users*	Educators, students, health care settings, pharmacologists, patients	Hotel management, anti-terrorism strategists, hotel employees	Clinicians, patients, students, researchers, insurance representatives	Clinicians, patients, students, researchers, insurance representatives
Type of purpose(s) (not mutually exclusive)	Policy development	Guidelines Policy development	Guidelines Classifications Policy development	Guidelines Classifications Policy development Current state of knowledge

**Table 3.** Four examples of expert consensus methods in humanities related research described by type.

Expert consensus method	Delphi study	Nominal group	Mixed and tailor-made method that somewhat resembles a consensus conference	Mixed and tailor-made method that somewhat resembles a Glaser’s state of the art method
**Publication**	( Lechner *et al.* 2023)	( Dragouni and Lekakis 2023)	( Rulkens *et al.* 2022-2023)	( *Standing Committee for the Museum Definition – ICOM Define Final Report (2020-2022)* 2022) and ( *ICOM Webinar Defining the Museum in Times of Change: A Way Forward *2020)
**Field**	Philosophy (Epistemology)	Conservation (Heritage management)	Visual Arts (Art History)	Museology
(i) *Process leaders or steering committee*	Authors of the paper	Authors of the paper	Multidisciplinary group of researchers	International Council of Museums (ICOM)
(ii) *Experts*	46 Researchers on epistemic responsibilities and/or university assessment instruments and/or (former) administrators with practical knowledge and hands-on experience in leading a university	32 Representatives of local government; academic researchers and members of cultural associations, artists, and residents	4 Rembrandt scholars with various kinds of expertise in art history and technical research into paintings	126 National Committees of museum professionals that are members of ICOM, (precise number of experts unknown)
(iii) *Issue*	What are the core epistemic responsibilities of universities?	What are solutions/actions and/or tactics to protect the rural monuments of the island of Naxos?	Are two paintings painted by Rembrandt, partially painted by Rembrandt or not painted by Rembrandt?	What should count as an international definition of the museum?
(iv) *Rules of engagemen*t (consensus threshold)	Three-round online survey, alternating between closed questions to gain consensus, and open questions to let experts motivate their answers. Panellists rated their agreement to consensus questions on a 5-point Likert scale (strongly agree – somewhat agree – neither agree nor disagree – somewhat disagree – strongly disagree). Consensus defined as 67% (i.e., two-thirds) of panellists (strongly) agreeing to a question. If no consensus was reached, the steering committee made a final decision	Panellists were divided in 5 groups which each met for 2 hours. Five main stages were completed: (i) presentation of problem-issue (ii) ideas sharing and generation and recording, (iii) discussion and listing, (iv) voting and (v) vote counting and conclusions. Consensus was establishes through majority and the qualitative generated data was thematically analysed	Panellists individually assessed the consensus questions and filled in a form in which they could address in percentages the extend in which they were sure about their answers. Subsequently, they were each interviewed to ask for motivations. This was followed by a focus group discussion, led by a chair. The experts then filled in a second form to examine if answers changed due to the group discussion. This was followed by an interview to ask about their motivations. The method ended with a joint debriefing. Qualitative data was thematically analysed. Consensus was defined as 75%, i.e. when three out of four experts (three-quarters) agreed to a question	Committees each shared max 100 keywords for the definition, after discussion each committee submitted max 20 keywords for the definition. Then the keywords were analysed externally and the results were published. Committees reviewed the published results and submitted comments. They could add up to 3 new keywords and comment on keywords they could not accept. A final list of keywords was compiled. Based on this, a small group of special define members submitted 14 proposals for the definition. 5 Were selected by them for publication. Committees identified their preferred proposal. This final proposal was published and put to vote during an international extraordinary general assembly of ICOM members
(v) *Output*	6 core epistemic responsibilities of universities	22 ideas reflecting current needs and proposing responding actions to safeguarding the future of the rural heritage of the island of Naxos	Full consensus amongst panellists about the attribution of two 17 ^th^ century paintings	An internationally supported definition of the museum
(vi) *Users*	Higher education policymakers and university leadership	Heritage experts, managers of rural resources, local communities	Specialists of 17 ^th^ century Dutch paintings, the museums that own these paintings, museum visitors	Museums, museum professionals, policymakers and governments world-wide
Type of purpose(s) (not mutually exclusive)	Policy development	Policy development Guidelines	Current state of knowledge	Taxonomies/definitions

**Table 4.** Examples of purposes for which expert consensus methods can be employed, partially based on Gattrell et al. (2024, Table 2).

Purpose:	How consensus helps, incorporating multiple perspectives and/or expertises:
Guidelines	Translating evidence into recommendations
Classifications	Definition of markers, signs or thresholds
Research priorities	Defining and ranking priorities in the context of limited resources
Policy development	Analysing and interpreting evidence to inform policies
Taxonomies/definitions	Defining standard taxonomies and definitions
Current state of knowledge	Defining the status quo of consensus or dissensus on a research topic

## 5. Expert consensus methods in the humanities: limitations and potential

In the preceding sections, we examined the elements and dynamics of expert consensus methods, along with examples and possibilities for intended objectives. In this section, we delve into the question why formal approaches to consensus in the humanities remain scarce, despite the significant factor of consensus and dissensus in knowledge production across research cultures. Aside from limitations, we also address what the potential added value of applying consensus methods to humanities-related issues could be.

First of all, practical limitations likely play a role. Expert consensus methods and their subsequent publication demand significant time and resources, which are rarely in abundance in the humanities. Additionally, a factor that may affect academia broadly is the emphasis on publication quantity and individual contributions, which can discourage scholars from lengthy endeavours for which recognition is shared ( Leising *et al.* 2024). Moreover, expert consensus methods are not interwoven in humanities research traditions. Researchers may not be familiar with consensus methods, and the advantages they may have for their area of research.

Secondly, concerns raised in the field of psychology that may apply in the humanities as well, highlight risks of consensus-building. Emphasizing consensus may lead to agreement for the wrong reasons, such as conformity, which may be harmful to innovation, creativity and minority views. It can sideline dissent, whilst that is a vital element in the evolution of science (Gollwitzer 2022; Asendorpf and Gebauer 2022; Beck *et al.* 2022; Denissen and Sijtsma 2022; Hilbig *et al.* 2022). Additionally, experts agreeing on something does not necessarily mean they are correct. They may be in full agreement on a point that is incorrect or untrue.

Scholars may also disagree due to the role of research paradigms or schools within the humanities, as researchers’ positionality can shape their interpretations. In addition to that, humanities research can be concerned with hermeneutics, i.e., issues that have to do with understanding the values and meanings of texts and other objects. And many humanities fields such as ethics, epistemology, and metaphysics, rely on a-priori methods like thought experiments, reasoning on the basis of intuitions and principles, and formal logic rather than empirical data. This allows for multiple interpretations that can all to some extent be warranted by the evidence base, and may not readily align into a singular consensus. These factors suggest that the complexity and interpretative nature of humanities research might render expert consensus methods to some extent less relevant.

Nevertheless, we believe that there are compelling reasons for considering expert consensus methods in humanities research. First of all, opposing the above concerns, one of the important purposes of expert consensus methods is to bring various viewpoints, nuances, and interpretations to the surface, so that they can be discussed. Depending on the design and purpose of the consensus method, it could reveal levels of consensus and/or dissensus on an issue, within or across schools of thought or paradigms. They might even have the potential to articulate both divergences and common grounds regarding an issue, and by extension increase mutual understanding.

The application of such a method may therefore stimulate epistemic progress in itself. Moreover, a carefully designed protocol and curated group of experts can foster a fair practice and ensure the consultation of diverse viewpoints. Expert consensus methods provide opportunities to mitigate biases and their ramifications by this cautious selection of experts, but also by blinding certain parts of the process in relation to anonymity of participating experts, for example.

Secondly, expert consensus methods can be helpful in getting a firmer grip on the status quo or state of the art on a particular issue in a field (e.g. Hodgkin *et al.* (1975) mentioned in Table 2; Rulkens *et al.* 2022-2023, mentioned in Table 3). Of course, scholars often already have an idea about that, and such an informed opinion is valuable in itself. However, it can be complemented by a more structured approach, particularly when the number of scholars involved is large. This approach can additionally provide guidance for future research by pointing out the gaps in the current evidence base. And when applied on a recurrent basis, it might even have the potential to gain insight in how views evolve over time. It is important to note that consensus is not a final endpoint; rather, it can be continually re-evaluated in light of new insights and reasonable doubts regarding its validity.

Third, an advantage of expert consensus methods is that they enhance the transparency and replicability of research because of their well-documented and systemised approach (for replication in the humanities, see Peels and Bouter (2018)). It can therefore be instrumental to epistemic progress beyond the consensus reached at a moment in time.

Fourth, application or implementation of its outcomes is often easier after the process of a consensus method is completed (e.g. Standing Committee for the Museum Definition (2020-2022) (2022), mentioned in Table 3). And finally, expert consensus methods can aid public understanding by clarifying experts perspectives on specific issues ( Stekelenburg *et al.* (2022) thereby enabling the public to make informed decisions ( Ordway 2021).

## 6. Conclusion

In this article, we have presented a framework comprising six key elements for the application of expert consensus methods, alongside examples that illustrate different types of these methods and their purposes in various research contexts. This serves as a preliminary exploration of the potential value these methods may offer for the humanities. Despite inevitable practical limitations, expert consensus methods can accelerate epistemic process, enhance transparency, increase replicability, stimulate diversity, and encourage fair processes in humanities research and the application of its findings. There seem to be humanities-related issues that may lend themselves better to structured expert consensus methods than their subject matter and research methods might suggest. However, applying a consensus method is a costly endeavour in terms of time and means. There might be conditions under which these efforts do not live up to the epistemic benefits they provide for. The identification of the particular areas and issues where these methods hold most promise can be done by assessing the purposes these methods have served, and translating those to a specific area of research. The assessment and prioritization of issues, especially when resources are limited, should be carried out locally by researchers who are familiar with the epistemologies and issues relevant to their field. Consequently, it is essential to carry out future research involving humanities case studies that employ expert consensus methods, to evaluate their feasibility and effectiveness. Collaboration between humanities researchers with scholars from other domains experienced in designing consensus methods could facilitate mutual learning and foster innovative approaches.

## Author contributions

Charlotte C.S. Rulkens and Rik Peels delivered the overall concept and text for this article, which Lidwine B. Mokkink, Tamarinde Haven and Lex Bouter critically read and revised. In addition to that, Tamarinde Haven and Lidwine B. Mokkink were responsible for Tables 1 and 2.

## Data Availability

Data sharing is not applicable to this article as no datasets were generated or analysed during the current study.

## Footnotes

^1^
We acknowledge that as is the case in the sciences, the humanities encompass a large amount of disciplines and subfields, exhibiting significant diversity in research types and methodologies. However, in this paper we refer to ‘the humanities’ to clarify difference for the reader, rather than out of belief in rigid categorizations. For readers interested in this topic, see e.g. Woudenberg (2018) and Grimm *et al.* ( *forthcoming*).
