# Supplementary material for: Expert Consensus Methods In The Humanities: An Exploration of their Potential
Source: F1000Res. 2024 Dec 16;13:710. Originally published 2024 Jun 28. [Version 2] doi: 10.12688/f1000research.148726.2 (PMC11754948; doi:10.12688/f1000research.148726.2)
Supplement: Peer review history

# Reviewer report: Reviewer response for version 2

**DOI:** 10.5256/f1000research.174565.r355056

**Published:** 2025-01-22

**Authors:** Stephen R Grimm

**Affiliations:** 1 Fordham University, Bronx, USA

This is a very nice, clear overview of the nature and potential importance of consensus methods in the humanities.  I especially appreciate the cautions at the end, namely that "applying a consensus method is a costly endeavour in terms of time and means. There might be conditions under which these efforts do not live up to the epistemic benefits they provide for."  I too wondered this throughout reading the paper.  But, that said, I can also see the benefits of pursuing these methods in the humanities.

Is the rationale for developing the new method (or application) clearly explained?

Yes

Is the description of the method technically sound?

Yes

Are the conclusions about the method and its performance adequately supported by the findings presented in the article?

Yes

If any results are presented, are all the source data underlying the results available to ensure full reproducibility?

Yes

Are sufficient details provided to allow replication of the method development and its use by others?

Yes

Reviewer Expertise:

Philosophy, epistemology

I confirm that I have read this submission and believe that I have an appropriate level of expertise to confirm that it is of an acceptable scientific standard.

# Reviewer report: Reviewer response for version 2

**DOI:** 10.5256/f1000research.174565.r349191

**Published:** 2025-01-09

**Authors:** Emanuela Reale

**Affiliations:** 1 CNR IRCRES—Research Institute for Sustainable Economic Growth, Rome, Italy

I have read the revised version of the paper and the changes that have been applied. Some of my suggestions have been addressed and the manuscript in the current form is clearer and more consistent than before. However, the manuscript still does not have a clear research question and therefore it remains largely descriptive and the scientific contribution it provides does not emerge. Problems and limitations with the applications of the consensus methods in the Humanities are not discussed with reference to the specific cases that have been presented in tables 3-4 and with empirical evidence on the cases themselves. Moreover, some limitations are not exclusive of Humanities (e.g. time and resources; role of research paradigms bringing to divergent views) and the specific features of Humanities would need a deeper analysis.

Is the rationale for developing the new method (or application) clearly explained?

No

Is the description of the method technically sound?

No

Are the conclusions about the method and its performance adequately supported by the findings presented in the article?

No

If any results are presented, are all the source data underlying the results available to ensure full reproducibility?

Yes

Are sufficient details provided to allow replication of the method development and its use by others?

No

Reviewer Expertise:

Higher education and research policy

I confirm that I have read this submission and believe that I have an appropriate level of expertise to confirm that it is of an acceptable scientific standard, however I have significant reservations, as outlined above.

# Reviewer report: Reviewer response for version 2

**DOI:** 10.5256/f1000research.174565.r349190

**Published:** 2024-12-23

**Authors:** Daniel Leising

**Affiliations:** 1 Technische Universitat Dresden, Dresden, Saxony, Germany

I found that the authors responded to my comments in a careful and throughtful manner, and that the manuscript has improved. I have no further comments or suggestions for changes.

Is the rationale for developing the new method (or application) clearly explained?

Yes

Is the description of the method technically sound?

Yes

Are the conclusions about the method and its performance adequately supported by the findings presented in the article?

Yes

If any results are presented, are all the source data underlying the results available to ensure full reproducibility?

No source data required

Are sufficient details provided to allow replication of the method development and its use by others?

Yes

Reviewer Expertise:

Personality / Social Psychology, Meta-Science

I confirm that I have read this submission and believe that I have an appropriate level of expertise to confirm that it is of an acceptable scientific standard.

# Reviewer report: Reviewer response for version 1

**DOI:** 10.5256/f1000research.163073.r309049

**Published:** 2024-09-19

**Authors:** Daniel Leising

**Affiliations:** 1 Technische Universitat Dresden, Dresden, Saxony, Germany

I strongly agree that more systematic consensus-building is needed in many fields of science, including the Humanities and Psychology (my own field) and I find little that I would disagree with in the manuscript as is.

 However, I also think that the authors could do more than just presenting an overview of established consensus building methods and suggesting that researchers from the Humanities should give these methods a try. The issue is more complex than that. In order to improve on the depth, utility and potential impact of this paper, I would thus like to encourage the authors to think about and – if possible – share their views on a number of additional, intriguing, and sometimes thorny issues regarding consensus building. 

 First, I would argue that the Humanities are actually not that “unique” in terms of their nature and approaches (page 11). To enable a proper discussion of this issue, the authors could explain their own view of what is so specific about the Humanities in a bit more detail.

 Here is my view: Interviews and text analyses are used in many different branches of science. Exploratory research is also conducted in many fields. The ways in which people give meaning to their experiences are also of interest across various branches of science. And even in many of the “harder” sciences, research is often “coloured by the backgrounds and positionality of the researchers” (page 8). So, much of what is common in research in the Humanities is actually shared with other fields. 

 In my view, two features may actually distinguish “the Humanities” from other fields - but even these distinctions are only gradual in nature. One feature is the common practice of using everyday language as the primary scientific language. The other feature is the relative fuzziness of many concepts - which I would note is not necessarily a weakness but may often be unavoidable given the complexity of the phenomena of interest (and also given the first feature just mentioned). Do the authors agree?  

 Second, I would like to encourage the authors to say a bit more about possible reasons for why so little consensus building is taking place in many fields of science, despite it being widely viewed as desirable. In my view, there are a couple of reasons, most of which are rather extraneous to an idealized image of science (as a collaborative and cumulative effort at building a reliable knowledge base for the good of mankind). 

 For example, consensus building is likely to reduce the publication output of a field, which would be at odds with the interests of commercial publishers. Also, individual researchers have an interest in “making a name for themselves" based on their specific expertise or views regarding certain topics. Consensus building makes it more difficult to maintain a highly distinct profile and thus reduce visibility.  This may be risky in a science system that is as competitive as the one we choose to entertain.  

 And then there may be a need to “save face”: The more of your career you have dedicated to certain ideas (e.g., your own theory of some phenomenon), the harder it may be to let those ideas go - even if the evidence against them is overwhelming. There is a reason why Max Planck once stated that scientific progress happens “one funeral at a time”.

 At least a brief nod to the relevance of such more political factors would be nice - if the authors share my conviction that they are relevant. Who has an actual interest in achieving consensus? Who has an interest in avoiding the emergence of consensus? And what are the external incentives for and agaisnt this type of work?   

 Third, I would also appreciate if the authors could talk some more about “bad consensus” – views that are held by many, despite their being wrong. With a particular eye to the Humanities – what criteria are there, apart from consensus, that may help us determine whether a view is “correct”? Or may truth in the Humanities *only* be defined in terms of relative consensus among researchers? In the harder sciences, the ability to *predict* the occurrence of certain events from a theory (and some data input) plays a crucial role as evidence of the theory's correctness. Are the Humanities able to produce testable predictions, too? Can there be consensus projects in the Humanities that actually test one theory against another? 

 Fourth, on page 3 the authors state that **“**Since the questions addressed in the humanities may leave room for multiple viewpoints that are equally warranted by the evidence base, it can be infeasible or not desirable to get a complete or almost complete consensus.” To this I would respond: what are the conditions under which working toward consensus would not be feasible, or desirable? I agree that this may be the case – but when is it the case, and when is it not the case? I personally hold the somewhat skeptical view that many researchers will shy away from working toward consensus even if doing so *would* be both feasible and desirable (see my previous point). Such a “live and let live” attitude may often be relatively comfortable to have (e.g., to avoid conflict). It may, however, be regarded as unethical, because it will foster the preservation of an unnecessary level of ambiguity. 

 Minor points

 Fifth, the authors state that a steering committee would typically be comprised of the people who initiated the process and who will ultimately author the consensus document. Could there not be cases in which the process should better be overseen by an independent third party (e.g., a moderator)? Especially when issues are highly contentious, for example? And should not all the experts who worked on the consensus be authors on the consensus document?  

 Sixth, something is not quite right on page 4, text lines 16-19. 

 Seventh, I would say that evidence will never “speak for itself” (page 4) - otherwise papers in certain scientific fields would not need any Discussion sections.

 Together with a variety of colleagues, I have written about several of the above-mentioned issues. If the authors are interested in this, here are a few key references: I referenced some of our own publications that I consider to be related in terms of content, to let the authors know about their existence. The authors are of course entirely free to decide whether to look into them, or not. 

 Leising, D.et.al; 2023 (Ref 1)

 Leising, D.et.al; 2024 (Ref 2)

 Leising, D.et.al; 2022 (Ref 3)

 Leising, D.et.al; 2022 (Ref 4)

Is the rationale for developing the new method (or application) clearly explained?

Yes

Is the description of the method technically sound?

Yes

Are the conclusions about the method and its performance adequately supported by the findings presented in the article?

Yes

If any results are presented, are all the source data underlying the results available to ensure full reproducibility?

No source data required

Are sufficient details provided to allow replication of the method development and its use by others?

Yes

Reviewer Expertise:

Personality / Social Psychology, Meta-Science

I confirm that I have read this submission and believe that I have an appropriate level of expertise to confirm that it is of an acceptable scientific standard, however I have significant reservations, as outlined above.

## References

[1] : Visual Argument Structure Tool (VAST) Version 1.0. *Meta-Psychology* .2023;7: 10.15626/MP.2021.2911 10.15626/MP.2021.2911

[2] : A Tentative Roadmap for Consensus-Building Processes.2024; 10.31234/osf.io/7t98r 10.31234/osf.io/7t98r

[3] : Ten steps toward a better personality science – how quality may be rewarded more in research evaluation. *Personality Science* .2022;3: 10.5964/ps.6029 10.5964/ps.6029

[4] : Ten steps toward a better personality science – a rejoinder to the comments. *Personality Science* .2022;3: 10.5964/ps.7961 10.5964/ps.7961

# Response

**Published:** 2024-11-09

**Authors:** Charlotte Rulkens

**Affiliations:** Philosophy, Vrije Universiteit Amsterdam, Amsterdam, North Holland, The Netherlands

Dear reviewers,

 We sincerely thank you for the thorough reviews of our manuscript titled *Expert Consensus Methods In The Humanities - An Exploration of their Potential. *We appreciate the time and effort you have put into evaluating our work and for providing insightful comments and valuable suggestions. We have carefully considered each of them and have revised the manuscript accordingly. Below, we provide detailed responses to each of the comments.

 We believe that the revisions have improved the manuscript, and we hope that our responses meet any remaining concerns.

**Response to reviewer 1: Emanuela Reale, CNR IRCRES—Research Institute for Sustainable Economic Growth, Rome, Italy**

**Reviewer 1, Comment 1: **First, the aim of the paper is clear, but the research question(s) related to that aim are missing. This is an important limitation, since the manuscript reports an exploratory effort and therefore a delimitation of the research problem that you want to investigate is very useful to delineate the perimeter of the work.

**Response: **We acknowledge the concern raised and have addressed this by rewriting the introduction section and by incorporating a clearer description of our study goal. We now make it more clear that our goal was primarily didactic, in the sense that we sought to explore the usefulness of consensus methods utilised in the natural, social, and life sciences for the humanities (see section 1, lines 59-83):

 An important way to make progress in knowledge and understanding is to gradually, via the exchange of evidence, expertise, and critical discussion, reach (more) consensus on a topic. However, whether there is consensus on a certain matter at a moment in time can sometimes remain unknown, be unclear, or remain implicit. Expert consensus methods can lift off this inconclusiveness by intentionally and formally pursuing or assessing consensus. These methods are regularly used in natural, social, and life sciences. Despite the significant role of consensus in the development of (academic) knowledge and understanding (e.g. Kuhn, 1962; Habermas, 1996; thus also Peels, 2020; Grimm et al., *forthcoming*), formal expert consensus methods have hardly been explored within in the context of the humanities. [1] Other kinds of valuable expert meetings do take place in various disciplines in the humanities. They usually do not seek to reach a consensus, but to explore a topic in-depth by bringing the relevant experts to the table. Moreover, expert consensus methods described in this article differ from the expert meetings that already take place in the humanities by the former’s procedural and formalised character.

 In this paper, we aim to highlight to scholars, particularly those in the humanities, what is possible in terms of a systematic approach to academic consensus. In doing so, we aim to initiate exploration of and discussion about the potential of applying such an approach. What would be the added value of applying expert consensus meetings to humanities-related issues? To achieve this, we first explicate our method (§2), before we provide a framework for the application of expert consensus methods by introducing six key elements (§3). Subsequently, we present common types of expert consensus methods utilized in the natural, social, and life sciences. We highlight their and proven value across various types of research through relevant examples and their respective objectives (§4). We then consider both the potential benefits and possible limitations to applying these methods within humanistic research (§5), and conclude with suggestions for future research (§6).

**Reviewer 1, Comment 2: **Second, the section on Method is poor. The composition of the interdisciplinary team (and the reasons for using this team) is not clearly described, the brainstorm session is not properly defined and illustrated, the ‘informal’ literature review is something new for me (what ‘informal’ means?) and it is not acceptable for a research paper to quote two methods used (‘philosophical methods of conceptual analysis’ and ‘conceptual engineering’) without explaining how they have been used for the purposes of the paper but just putting two references. Also, the process of writing and reviewing the manuscript should be better described and justified.

**Response: **Our article, as we now emphasise more, is meant as an exploratory comment and a didactic contribution to inspire scholars in the humanities to see whether consensus methods may be valuable within their own discipline or sub discipline. It is not an instance of systematic empirical research, which would have required a much more detailed methodological section. But we certainly we agree that the method section can be improved in various ways. Therefore, we extended the section by including a specification of the backgrounds of the involved authors and specified the process of our collaboration as follows (see section 2, lines 85-94):

 To explore the potential of applying expert consensus methods in the humanities, we composed an interdisciplinary team of both researchers from the sciences using consensus methods in practice (LM: epidemiology; TH: qualitative research methodology; LB: epidemiology and data science, research integrity), and researchers familiar with the domain of the humanities and epistemology (CR: art history, museology; RP: epistemology and philosophy of science, religion and theology). In a first brainstorm session, knowledge on the topic was shared from both perspectives. This resulted in a list of consensus methods and examples used in the sciences, drafted by LM and TH. CR carried out a literature search in order to find examples thereof that were applied in her domain. A first draft was written and discussed in a second group session and culminated in a final version, which was edited and reviewed by all authors.

**Reviewer 1, Comment 3: **Third, and more important, the paper refers always to ‘humanities.’ Humanities is a highly fragmented and differentiated field, where many and different epistemic communities work, interact, shape their own idea and uses of consensus method (if any) and where different instances of relationships with other researchers within the field and with neighboring fields are at place. The paper does not acknowledge this important characteristic of humanities (see footnote 1 where there is a list of disciplines included in the humanities that are considered in the paper). The same holds true for STEMs, which are considered as they are all adopting regularly expert consensus methods and there is no explanation or evidence about it. In sum, the paper ‘explores’ the expert consensus method without considering the set of ideas, values, theories, concepts and practices that pertain to the disciplines analyzed and their scientific work.

**Response: **We acknowledge the concern raised and have taken this into account by adjusting a footnote 1 in section 1 (see above) and by revising section 4. Upon reflection we furthermore adjusted the ways to delineate the contexts in which expert consensus methods may be valuable, by focusing on one’s purpose in applying such a method (see section 4, lines 213-235):

 The subsequent question would be to determine the contexts in which this approach to consensus would prove most valuable. Research in both the humanities and the sciences spans a wide array of disciplines and sub disciplines, with a vast range of variation in types of research and methods, even within the most specialised areas. Classification of research into one of these domains is often gradual in nature, especially since interdisciplinary research increasingly combines approaches traditionally linked to the ‘humanities’ or ‘sciences’. Making general statements on the types of research in which expert consensus methods might be valuable related to the ‘humanities’ versus the ‘sciences’ is therefore difficult. We decided to examine objectives for employing consensus methods in various contexts in which they have demonstrated their value. This could contribute to a directory of instances in which these methods may prove useful in humanities-related fields. Gattrell et al. (2024, Table 2) present examples of these objectives in context of the health sciences, such as the establishment of clinical practice guidelines, diagnostic guidelines or reporting guidelines but also the classification of diseases, the setting research priorities, or the formulation of policy. These aims are summarised, adjusted and extended in Table 4 in a manner that enhances their generalisability to other fields of research and were included in Table 2 and 3. Although these examples highlight specific applications, we think that there is a broader potential for its use in the humanities. For instance, in establishing consensus and guidelines on the best way to preserve certain historical objects (guidelines/ policy development), involving eyewitnesses in the reconstruction of an historical event (current state of knowledge), or consulting various experts in establishing how to interpret an ancient source (current state of knowledge). Future research and case studies exploring the potential applications outlined in Table 4 could provide insights into specific instances where the pursuit of formal dis- or consensus may prove effective.

**Reviewer 1, Comment 4: **Fourth, sections 3 and 4 provide a clear summary of what expert consensus methods are and a deepen analysis of four types. Then the reader expects Section 5 discusses the results with respect to the exploration done. On the contrary, Section 5 lists of pros and cons related to the expert consensus method, and some examples of applications; the contents are completely unrelated with what the sections 3 and 4 have presented and the motivations for considering the expert consensus method an interesting development for humanities are unclear. Here the suggestion is to revise the sections trying to answer the research questions (when formulated) and highlight what we have learnt from the exploratory work, what is the contribution of the brainstorming to the exploration, what new avenues of research can be envisaged based on the results obtained, and what limitations/obstacles can the expert consensus model have in the humanities with respect to the other fields.

**Response: **Thank you for pointing this out to us. Based on this valuable feedback, we have worked to better align section 5 with the preceding sections to create a more cohesive flow throughout the paper and have made more explicit what our line of reasoning is. However, because the paper is intended as preliminary exploration, part of our manuscript aims to identify the potential value of these methods for the humanities. Section 5 is dedicated to that purpose and we made that more explicit in the revision (see section 5, lines 253-315):

 In the preceding sections, we examined the elements and dynamics of expert consensus methods, along with examples and possibilities for intended objectives. In this section, we delve into the question why formal approaches to consensus in the humanities remain scarce, despite the significant factor of consensus and dissensus in knowledge production across research cultures. Aside from limitations, we also address what the potential added value of applying consensus methods to humanities-related issues could be.

 First of all, practical limitations likely play a role. Expert consensus methods and their subsequent publication demand significant time and resources, which are rarely in abundance in the humanities. Additionally, a factor that may affect academia broadly is the emphasis on publication quantity and individual contributions, which can discourage scholars from lengthy endeavours for which recognition is shared (Leising et al., 2024). Moreover, expert consensus methods are not interwoven in humanities research traditions. Researchers may not be familiar with consensus methods, and the advantages they may have for their area of research.

 Secondly, concerns raised in the field of psychology that may apply in the humanities as well, highlight risks of consensus-building. Emphasising consensus may lead to agreement for the wrong reasons, such as conformity, which may be harmful to innovation, creativity and minority views. It can sideline dissent, whilst that is a vital element in the evolution of science (Gollwitzer, 2022; Asendorpf & Gebauer, 2022; Beck et al., 2022; Denissen & Sijtsma, 2022; Hilbig et al., 2022). Additionally, experts agreeing on something does not necessarily mean they are correct. They may be in full agreement on a point that is incorrect or untrue.

 Scholars may also disagree due to the role of research paradigms or schools within the humanities, as researchers’ positionality can shape their interpretations. In addition to that, humanities research can be concerned with hermeneutics, i.e., issues that have to do with understanding the values and meanings of texts and other objects. And many humanities fields such as ethics, epistemology, and metaphysics, rely on apriori methods like thought experiments, reasoning on the basis of intuitions and principles, and formal logic rather than empirical data. This allows for multiple interpretations that can all to some extent be warranted by the evidence base, and may not readily align into a singular consensus. These factors suggest that the complexity and interpretative nature of humanities research might render expert consensus methods to some extent less relevant.

 Nevertheless, we believe that there are compelling reasons for considering expert consensus methods in humanities research. First of all, opposing the above concerns, one of the important purposes of expert consensus methods is to bring various viewpoints, nuances, and interpretations to the surface, so that they can be discussed. Depending on the design and purpose of the consensus method, it could reveal levels of consensus and/or dissensus on an issue, within or across schools of thought or paradigms. They might even have the potential to articulate both divergences and common grounds regarding an issue, and by extension increase mutual understanding. 

  The application of such a method may therefore stimulate epistemic progress in itself. Moreover, a carefully designed protocol and curated group of experts can foster a fair practice and ensure the consultation of diverse viewpoints. Expert consensus methods provide opportunities to mitigate biases and their ramifications by this cautious selection of experts, but also by blinding certain parts of the process in relation to anonymity of participating experts, for example.

 Secondly, expert consensus methods can be helpful in getting a firmer grip on the status quo or state of the art on a particular issue in a field (e.g. Hodgkin et al. (1975) mentioned in Table 2; Rulkens et al. 2022-2023, mentioned in Table 3). Of course, scholars often already have an idea about that, and such an informed opinion is valuable in itself. However, it can be complemented by a more structured approach, particularly when the number of scholars involved is large. This approach can additionally provide guidance for future research by pointing out the gaps in the current evidence base. And when applied on a recurrent basis, it might even have the potential to gain insight in how views evolve over time. It is important to note that consensus is not a final endpoint; rather, it can be continually re-evaluated in light of new insights and reasonable doubts regarding its validity.

 Third, an advantage of expert consensus methods is that they enhance the transparency and replicability of research because of their well-documented and systematised approach (for replication in the humanities, see Peels and Bouter (2018)). It can therefore be instrumental to epistemic progress beyond the consensus reached at a moment in time.

 Fourth, application or implementation of its outcomes is often easier after the process of a consensus method is completed (e.g. Standing Committee for the Museum Definition (2020-2022) (2022), mentioned in Table 3). And finally, expert consensus methods can aid public understanding by clarifying experts perspectives on specific issues (Stekelenburg et al., 2022) thereby enabling the public to make informed decisions (Ordway, 2021).

 In response to this comments, we furthermore extended suggestions for future research in our conclusion (see section 5, lines 325-335):

 However, applying a consensus method is a costly endeavour in terms of time and means. There might be conditions under which these efforts do not live up to the epistemic benefits they provide for. The identification of the particular areas and issues where these methods hold most promise can be done by assessing the purposes these methods have served, and translating those to a specific area of research. The assessment and prioritisation of issues, especially when resources are limited, should be carried out locally by researchers who are familiar with the epistemologies and issues relevant to their field. Consequently, it is essential to carry out future research involving humanities case studies that employ expert consensus methods, to evaluate their feasibility and effectiveness. Collaboration between humanities researchers with scholars from other domains experienced in designing consensus methods could facilitate mutual learning and foster innovative approaches.

**Response to reviewer 2:** **Daniel Leising, Technische Universitat Dresden, Dresden, Saxony, Germany**

**Reviewer 2, Comment 1**: First, I would argue that the Humanities are actually not that “unique” in terms of their nature and approaches (page 11). To enable a proper discussion of this issue, the authors could explain their own view of what is so specific about the Humanities in a bit more detail. Here is my view: Interviews and text analyses are used in many different branches of science. Exploratory research is also conducted in many fields. The ways in which people give meaning to their experiences are also of interest across various branches of science. And even in many of the “harder” sciences, research is often “coloured by the backgrounds and positionality of the researchers” (page 8). So, much of what is common in research in the Humanities is actually shared with other fields.

 In my view, two features may actually distinguish “the Humanities” from other fields - but even these distinctions are only gradual in nature. One feature is the common practice of using everyday language as the primary scientific language. The other feature is the relative fuzziness of many concepts - which I would note is not necessarily a weakness but may often be unavoidable given the complexity of the phenomena of interest (and also given the first feature just mentioned). Do the authors agree? 

**Response: **Thank you for challenging us on this point. We agree that the borders between the general fields of the natural, social, and life sciences, and the humanities are fuzzy, when it comes to their objects, purposes, and methods. Whether there is something distinctive about the humanities – we believe that is compatible with fuzziness at the boarders – is a matter that we cannot possibly explore in this article (one of us does so in a book entitled *A Philosophy of the Humanities* that is forthcoming in 2025 with Oxford University Press, a volume co-authored by Stephen Grimm, Rik Peels, and René van Woudenberg). We *do* believe though that ordinary language use is not generally characteristic of the humanities (think of the highly technical language of metaphysics and epistemology), nor do we believe that insufficient clarity about core concepts is characteristic of the humanities.

             We agree though with the idea, also drawn to attention to by reviewer 1 in comment 3, that the humanities are a highly diversified field. We included that by adjusting footnote 1 in section 1:

 We acknowledge that as is the case in the sciences, the humanities encompass a large amount of disciplines and sub fields, exhibiting significant diversity in research types and methodologies. However, in this paper we refer to ‘the humanities’ to clarify difference for the reader, rather than out of belief in rigid categorisations. For readers interested in this topic, see e.g. Woudenberg (2018) and Grimm et al., ( *forthcoming*).

 Additionally, to be able to demarcate between contexts in which the formal approach might be beneficial, we decided to put an emphasis on the purposes for which these methods are employed in other domains. This would be a way in which researchers with expertise in their fields could identify opportunities for a formal approach to consensus (see section 4, lines 213-235, also quoted above in response to comment 3 of reviewer 1):

 The subsequent question would be to determine the contexts in which this approach to consensus would prove most valuable. Research in both the humanities and the sciences spans a wide array of disciplines and sub disciplines, with a vast range of variation in types of research and methods, even within the most specialised areas. Classification of research into one of these domains is often gradual in nature, especially since interdisciplinary research increasingly combines approaches traditionally linked to the ‘humanities’ or ‘sciences’. Making general statements on the types of research in which expert consensus methods might be valuable related to the ‘humanities’ versus the ‘sciences’ is therefore difficult. We decided to examine objectives for employing consensus methods in various contexts in which they have demonstrated their value. This could contribute to a directory of instances in which these methods may prove useful in humanities-related fields. Gattrell et al. (2024, Table 2) present examples of these objectives in context of the health sciences, such as the establishment of clinical practice guidelines, diagnostic guidelines or reporting guidelines but also the classification of diseases, the setting research priorities, or the formulation of policy. These aims are summarised, adjusted and extended in Table 4 in a manner that enhances their generalisability to other fields of research and were included in Table 2 and 3. Although these examples highlight specific applications, we think that there is a broader potential for its use in the humanities. For instance, in establishing consensus and guidelines on the best way to preserve certain historical objects (guidelines/ policy development), involving eyewitnesses in the reconstruction of an historical event (current state of knowledge), or consulting various experts in establishing how to interpret an ancient source (current state of knowledge). Future research and case studies exploring the potential applications outlined in Table 4 could provide insights into specific instances where the pursuit of formal dis- or consensus may prove effective.

**Reviewer 2, Comment 2: **Second, I would like to encourage the authors to say a bit more about possible reasons for why so little consensus building is taking place in many fields of science, despite it being widely viewed as desirable. In my view, there are a couple of reasons, most of which are rather extraneous to an idealised image of science (as a collaborative and cumulative effort at building a reliable knowledge base for the good of mankind). For example, consensus building is likely to reduce the publication output of a field, which would be at odds with the interests of commercial publishers. Also, individual researchers have an interest in “making a name for themselves” based on their specific expertise or views regarding certain topics. Consensus building makes it more difficult to maintain a highly distinct profile and thus reduce visibility.  This may be risky in a science system that is as competitive as the one we choose to entertain. And then there may be a need to “save face”: The more of your career you have dedicated to certain ideas (e.g., your own theory of some phenomenon), the harder it may be to let those ideas go - even if the evidence against them is overwhelming. There is a reason why Max Planck once stated that scientific progress happens “one funeral at a time”. At least a brief nod to the relevance of such more political factors would be nice - if the authors share my conviction that they are relevant. Who has an actual interest in achieving consensus? Who has an interest in avoiding the emergence of consensus? And what are the external incentives for and against this type of work **?**

**Response: **We think that these are valid points and the reviewers’ publications on the topic shed important light on this. We agree that external incentives may work against a formal approach to consensus as follows and have therefore slightly revised the manuscript on this point. We remain rather brief, though, as the exact explanation of why consensus methods are still relatively rarely used even in the sciences is an issue that is beyond the scope of our paper (see section 5, lines 259-264, also quoted in response to comment 4 of reviewer 1):

 Expert consensus methods and their subsequent publication demand significant time and resources, which are rarely in abundance in the humanities. Additionally, a factor that may affect academia broadly is the emphasis on publication quantity and individual contributions, which can discourage scholars from lengthy endeavours for which recognition is shared (Leising et al., 2024). Moreover, expert consensus methods are not interwoven in humanities research traditions. Researchers may not be familiar with consensus methods, and the advantages they may have for their area of research.

**Reviewer 2, Comment 3: **Third, I would also appreciate if the authors could talk some more about “bad consensus” – views that are held by many, despite their being wrong. With a particular eye to the Humanities – what criteria are there, apart from consensus, that may help us determine whether a view is “correct”? Or may truth in the Humanities *only* be defined in terms of relative consensus among researchers? In the harder sciences, the ability to *predict* the occurrence of certain events from a theory (and some data input) plays a crucial role as evidence of the theory's correctness. Are the Humanities able to produce testable predictions, too? Can there be consensus projects in the Humanities that actually test one theory against another? 

**Response: **This is an interesting point, that would need a elaborative reflection in order to do justice to the complexity of this issue. It is important to note, that we do believe that consensus and truth are two different things, and we made this more clear in our revised paper. Based these suggestions we also incorporated that consensus can be reached based on the wrong reasons and (see section 5, lines 265-271, also quoted in response to comment 4 of reviewer 1):

 Secondly, concerns raised in the field of psychology that may apply in the humanities as well, highlight risks of consensus-building. Emphasising consensus may lead to agreement for the wrong reasons, such as conformity, which may be harmful to innovation, creativity and minority views. It can sideline dissent, whilst that is a vital element in the evolution of science (Gollwitzer, 2022; Asendorpf & Gebauer, 2022; Beck et al., 2022; Denissen & Sijtsma, 2022; Hilbig et al., 2022). Additionally, experts agreeing on something does not necessarily mean they are correct. They may be in full agreement on a point that is incorrect or untrue.

**Reviewer 2, Comment 4: **Fourth, on page 3 the authors state that “Since the questions addressed in the humanities may leave room for multiple viewpoints that are equally warranted by the evidence base, it can be infeasible or not desirable to get a complete or almost complete consensus.” To this I would respond: what are the conditions under which working toward consensus would not be feasible, or desirable? I agree that this may be the case – but when is it the case, and when is it not the case? I personally hold the somewhat sceptical view that many researchers will shy away from working toward consensus even if doing so *would* be both feasible and desirable (see my previous point). Such a “live and let live” attitude may often be relatively comfortable to have (e.g., to avoid conflict). It may, however, be regarded as unethical, because it will foster the preservation of an unnecessary level of ambiguity. 

**Response:** We appreciate this insightful suggestion. Our approach to expert consensus methods does not necessarily imply working towards consensus in all instances, but is also related to the assessment of the (lack of) consensus. We view measuring consensus as a means, not an objective in itself. Furthermore, as put forward in paragraph 3, the bar for ‘sufficient’ consensus is something consensus researchers can determine their selves. Applying a consensus methods is a costly endeavour in terms of time and means. There might be conditions under which the effort of applying such a method does not live up to the epistemic benefits it provides for. We furthermore believe that the feasibility and desirability of assessing consensus is an issue that merits further exploration and included this as follows (see section 5, lines 325-335):

 However, applying a consensus method is a costly endeavour in terms of time and means. There might be conditions under which these efforts do not live up to the epistemic benefits they provide for. The identification of the particular areas and issues where these methods hold most promise can be done by assessing the purposes these methods have served, and translating those to a specific area of research. The assessment and prioritisation of issues, especially when resources are limited, should be carried out locally by researchers who are familiar with the epistemologies and issues relevant to their field. Consequently, it is essential to carry out future research involving humanities case studies that employ expert consensus methods, to evaluate their feasibility and effectiveness. Collaboration between humanities researchers with scholars from other domains experienced in designing consensus methods could facilitate mutual learning and foster innovative approaches.

**Minor comments:**

**Reviewer 2, Comment 5:**

 Fifth, the authors state that a steering committee would typically be comprised of the people who initiated the process and who will ultimately author the consensus document. Could there not be cases in which the process should better be overseen by an independent third party (e.g., a moderator)? Especially when issues are highly contentious, for example? And should not all the experts who worked on the consensus be authors on the consensus document?  

**Response: **Thank you for this relevant point, this is certainly the case and we made this more clear in the revised manuscript by the following addition (see section 3, lines 193-198):

 Naturally, the six elements can be specified at by the steering committee. If they deem it valuable to include an independent moderator or to incorporate a member with a critical or divergent perspective—especially if the topic is highly controversial—this remains within their discretion. Additionally, it is up to them to decide who will be listed as co-authors, whether that includes only the steering committee, all participants, or a broader group representation such as the panellists as a whole, for instance.

**Reviewer 2, Comment 6:**

 Sixth, something is not quite right on page 4, text lines 16-19. 

**Response:**

 Thank you for noticing, we have corrected this sentence as follows (see section 3, lines 136-140):

 Expert consensus methods are (i) applied by a *process leader or steering committee*, (ii) to reach consensus or assess the degree of consensus among a group of *experts*, (iii) about an *issue*, (iv) based on preset *rules of engagement*, (v) with the aim to deliver useful *output*, (vi) for future *users*. They are characterised by constructive and procedural usage of (dis)agreement and the different arguments that are provided therein.

**Reviewer 2, Comment 7:**

 Seventh, I would say that evidence will never “speak for itself” (page 4) - otherwise papers in certain scientific fields would not need any Discussion sections.

**Response: **Thank you for rightly pointing this out to us. We adjusted this as follows (see section 3, lines 132-134):

 This can be useful when the issue at hand is an important one and when evidence is lacking, it is inconsistent, or its interpretation is not obvious.

 As a result of our reactions to the comments, we also made adjustments to our abstract and conclusion, which are not all quoted in the above letter. We hope these revisions and clarifications meet the reviewers’ expectations. Please let us know if any further revisions are needed.

 Sincerely,

 Charlotte Rulkens MA

 Prof. dr. Rik Peels

 Dr. Lidwine Mokkink

 Dr. Tamarinde Haven

 Prof. em. dr. Lex Bouter

[1] We acknowledge that as is the case in the sciences, the humanities encompass a large amount of disciplines and sub fields, exhibiting significant diversity in research types and methodologies. However, in this paper we refer to ‘the humanities’ to clarify difference for the reader, rather than out of belief in rigid categorisations. For readers interested in this topic, see e.g. Woudenberg (2018) and Grimm et al., ( *forthcoming*).

# Reviewer report: Reviewer response for version 1

**DOI:** 10.5256/f1000research.163073.r296789

**Published:** 2024-08-23

**Authors:** Emanuela Reale

**Affiliations:** 1 CNR IRCRES—Research Institute for Sustainable Economic Growth, Rome, Italy

Expert Consensus Methods In The Humanities – An Exploration of their Potential

 The paper wants to explore the value of applying expert consensus methods in the humanities. The topic is very interesting and deserve attention. The manuscript is clearly written, and the literature mobilized is appropriate and relevant for the aim of the investigation proposed. The effort of synthetize concepts and findings using tables is valuable for the clarity of the argument. However, the manuscript in its current form has several weaknesses and, in my opinion, needs some substantial re-work. Hereby I try to summarize the most important critical issues that must be solved.

 First, the aim of the paper is clear, but the research question(s) related to that aim are missing. This is an important limitation, since the manuscript reports an exploratory effort and therefore a delimitation of the research problem that you want to investigate is very useful to delineate the perimeter of the work.

 Second, the section on Method is poor. The composition of the interdisciplinary team (and the reasons for using this team) is not clearly described, the brainstorm session is not properly defined and illustrated, the ‘informal’ literature review is something new for me (what ‘informal’ means?) and it is not acceptable for a research paper to quote two methods used (‘philosophical methods of conceptual analysis’ and ‘conceptual engineering’) without explaining how they have been used for the purposes of the paper but just putting two references. Also, the process of writing and reviewing the manuscript should be better described and justified.

 Third, and more important, the paper refers always to ‘humanities.’ Humanities is a highly fragmented and differentiated field, where many and different epistemic communities work, interact, shape their own idea and uses of consensus method (if any) and where different instances of relationships with other researchers within the field and with neighboring fields are at place. The paper does not acknowledge this important characteristic of humanities (see footnote 1 where there is a list of disciplines included in the humanities that are considered in the paper). The same holds true for STEMs, which are considered as they are all adopting regularly expert consensus methods and there is no explanation or evidence about it. In sum, the paper ‘explores’ the expert consensus method without considering the set of ideas, values, theories, concepts and practices that pertain to the disciplines analyzed and their scientific work.

 Fourth, sections 3 and 4 provide a clear summary of what expert consensus methods are and a deepen analysis of four types. Then the reader expects Section 5 discusses the results with respect to the exploration done. On the contrary, Section 5 lists of pros and cons related to the expert consensus method, and some examples of applications; the contents are completely unrelated with what the sections 3 and 4 have presented and the motivations for considering the expert consensus method an interesting development for humanities are unclear.

 Here the suggestion is to revise the sections trying to answer the research questions (when formulated) and highlight what we have learnt from the exploratory work, what is the contribution of the brainstorming to the exploration, what new avenues of research can be envisaged based on the results obtained, and what limitations/obstacles can the expert consensus model have in the humanities with respect to the other fields.

Is the rationale for developing the new method (or application) clearly explained?

No

Is the description of the method technically sound?

No

Are the conclusions about the method and its performance adequately supported by the findings presented in the article?

No

If any results are presented, are all the source data underlying the results available to ensure full reproducibility?

Yes

Are sufficient details provided to allow replication of the method development and its use by others?

No

Reviewer Expertise:

Higher education and research policy

I confirm that I have read this submission and believe that I have an appropriate level of expertise to state that I do not consider it to be of an acceptable scientific standard, for reasons outlined above.

# Response

**Published:** 2024-09-12

**Authors:** Charlotte Rulkens

**Affiliations:** Philosophy, Vrije Universiteit Amsterdam, Amsterdam, North Holland, The Netherlands

We sincerely thank the reviewer for her valuable comments, all of which we will address in the revision of our paper. We plan to begin this revision as soon as we receive the second review.

 We agree with the first and second suggestions to clarify our research questions, as well as the opportunities mentioned for improving the method section. While our method section may differ from those typically found in research papers, it is important to note that this paper is intended as a perspective piece with a didactic focus. It is aimed at highlighting to humanities scholars what is possible in terms of a systematic approach to academic ​​consensus on a topic and to initiate exploration of and discussion about the possible value of applying such an approach. Regarding the third comment, we will ensure that we address the issue of differentiation within research fields and discuss its implications for the application of consensus methods. Concerning the fourth point, we will work to better integrate section 5 with the preceding sections to create a more cohesive flow throughout the paper.
